# Supplementary material for: Implementing a choice of pulmonary rehabilitation models in chronic obstructive pulmonary disease (HomeBase2 trial): protocol for a cluster randomised controlled trial
Source: BMJ Open. 2022 Apr 10;12(4):e057311. doi: 10.1136/bmjopen-2021-057311 (PMC9003613; doi:10.1136/bmjopen-2021-057311)
Supplement: Author's manuscript [file bmjopen-2021-057311.draft_revisions.pdf]

BMJ Open is committed to open peer review. As part of this commitment we make the peer review history of every article we publish publicly available.

When an article is published we post the peer reviewers' comments and the authors' responses online. We also post the versions of the paper that were used during peer review. These are the versions that the peer review comments apply to.

The versions of the paper that follow are the versions that were submitted during the peer review process. They are not the versions of record or the final published versions. They should not be cited or distributed as the published version of this manuscript.

BMJ Open is an open access journal and the full, final, typeset and author-corrected version of record of the manuscript is available on our site with no access controls, subscription charges or pay-per-view fees (http://bmjopen.bmj.com).

If you have any questions on BMJ Open's open peer review process please email info.bmjopen@bmj.com

BMJ Open

Implementing a choice of pulmonary rehabilitation models in COPD (HomeBase2 trial): protocol for a cluster randomised controlled trial

Journal:	*BMJ Open*
Manuscript ID	bmjopen-2021-057311
Article Type:	Protocol
Date Submitted by the Author:	11-Sep-2021

Complete List of Authors:	Holland, Anne E.; Monash University; Institute for Breathing and Sleep<br>Jones, Arwel ; Monash University, Department of Allergy, Immunology and Respiratory Medicine<br>Mahal, Ajay; The University of Melbourne School of Population and Global Health<br>Lannin, Natasha; Monash University Faculty of Medicine Nursing and Health Sciences, Neuroscience<br>Cox, Narelle; Monash University , Allergy, Immunology and Respiratory Medicine; Institute for Breathing and Sleep<br>Hepworth, Graham; The University of Melbourne, Statistical Consulting Centre<br>O'Halloran, Paul; La Trobe University<br>McDonald, Christine; Austin Health, Respiratory and Sleep Medicine

Keywords:	Chronic airways disease < THORACIC MEDICINE, REHABILITATION MEDICINE, Emphysema < THORACIC MEDICINE

SCHOLARONE™
Manuscripts

Implementing a choice of pulmonary rehabilitation models in COPD (HomeBase2 trial): protocol for a cluster randomised controlled trial

Anne E Holland[1,2,3], Arwel W Jones[1], Ajay Mahal[4], Natasha A. Lannin[5,6] Narelle S Cox[1,3], Graham Hepworth[7], Paul O'Halloran[8], Christine F McDonald[9,10]

1. Respiratory Research @ Alfred, Monash University, Melbourne, Australia
2. Department of Physiotherapy, Alfred Health, Melbourne, Australia
3. Institute for Breathing and Sleep, Melbourne, Australia
4. The Nossal Global Institute for Global Health, University of Melbourne, Melbourne, Australia
5. Alfred Health, Melbourne, Victoria, Australia
6. Department of Clinical Neuroscience, Central Clinical School, Monash University, Melbourne, Australia
7. Statistical Consulting Centre, University of Melbourne, Melbourne, Australia
8. Department of Public Health, La Trobe University, Melbourne, Australia
9. Department of Respiratory and Sleep Medicine, Austin Health, Melbourne
10. Department of Medicine, University of Melbourne, Melbourne, Australia

Correspondence to: Prof Anne E Holland; a.holland@alfred.org.au

Authors' contributions

Study conception: AEH, CFM, AM, NAL, NSC; Development of protocol and methods: all authors; Review of manuscript and approval of final submitted version: all authors.

Funding statement

This work is supported by the Commonwealth of Australia Medical Research Future Fund grant MRF1176491. This funding source had no role in the design of this study and will not have any role during its execution, analyses, interpretation of the data, or decision to submit results.

Competing interests

AEH, AM, CFM, GH, NAL, NSC and PO declare grant funding from the Commonwealth of Australia Medical Research Future Fund paid to their institution for the conduct of the trial. CFM declares non-financial board roles as Chair COPD National Program, Lung Foundation Australia and Medical Director Institute for Breathing and Sleep. No other competing interests are declared.

For peer review only - http://bmjopen.bmj.com/site/about/guidelines.xhtml

Abstract

Introduction: Despite compelling evidence for centre-based pulmonary rehabilitation in the management of COPD, barriers at a health service and individual level prevent potentially eligible patients from accessing the benefits of this treatment. The aim of this implementation trial is to determine the clinical benefits and health care costs of offering patients a choice of two pulmonary rehabilitation locations (home or centre) compared to offering only the traditional centre-based model.

Methods and analysis: This two-arm cluster randomised, controlled, assessor-blinded trial conducted in 14 centre-based pulmonary rehabilitation services across Australia will recruit 490 participants with COPD. Pulmonary rehabilitation services will be randomised 1:1 to intervention (offering choice of home-based or centre-based pulmonary rehabilitation) or control (continuing to offer centre-based pulmonary rehabilitation only) sites stratified by centre-based program setting (hospital vs non-hospital). At both intervention and control sites, centre-based pulmonary rehabilitation will be delivered according to current best practice guidelines including supervised exercise training for 8 weeks. At intervention sites, home-based pulmonary rehabilitation will be delivered according to an established 8-week model, comprising of one home visit, unsupervised exercise training, and telephone calls that build motivation for exercise participation and facilitate self-management. The primary outcome is all-cause, non-elective hospitalisations in the 12 months following rehabilitation. Secondary outcomes include program completion rates and measurements of six-minute walk distance, chronic respiratory questionnaire, EQ-5D-5L and Dyspnoea-12 at the end of

For peer review only

rehabilitation and 12 months following rehabilitation. Direct healthcare costs, indirect costs and changes in EQ-5D-5L will be utilised to evaluate cost-effectiveness.

Ethics and dissemination: Alfred Hospital Human Research Ethics Committee has approved this protocol. The trial findings will be published in peer-reviewed journals, submitted to national or international conferences for presentation and disseminated to patients across Australia with support from Lung Foundation Australia and the Thoracic Society of Australia and New Zealand.

Trial registration number: NCT04217330

Strengths and limitations of this study

- This implementation trial, conducted across 14 sites, will be the first to compare the choice of home-based or centre-based pulmonary rehabilitation to centre-based pulmonary rehabilitation only.
- The trial will only include participants with a diagnosis of COPD.
- All participants, including those in the control group, will have the opportunity to achieve clinical benefits from an evidence-based pulmonary rehabilitation program.
- Participants will be blinded to group allocation and overall study aims but it is possible that the offer of choice of pulmonary rehabilitation location after consent is obtained at intervention sites will not reach patients who have strong preferences for home-based pulmonary rehabilitation before the offer.
- The primary outcome is hospitalisation, an outcome most valued by patients with COPD.

For peer review only - http://bmjopen.bmj.com/site/about/guidelines.xhtml

Introduction

Chronic obstructive pulmonary disease (COPD) is characterised by persistent respiratory symptoms and airflow limitation.[1] Activity-related dyspnoea and fatigue in COPD ultimately lead to reductions in physical activity and conditioning, which are associated with poor quality of life and exacerbations of respiratory symptoms.[2,3] Acute exacerbations are a leading cause of preventable hospitalisations and account for more than 50% of the costs associated with the treatment of COPD.[4,5,6] The goals of COPD management are to reduce exacerbations and hospitalisations by improving symptoms, optimising self-care and minimising disease progression.[1]

Pulmonary rehabilitation is defined as “a comprehensive intervention based on a thorough patient assessment followed by patient-tailored therapies that include, but are not limited to, exercise training, education, and behaviour change, designed to improve the physical and psychological condition of people with chronic respiratory disease and to promote the long-term adherence to health-enhancing behaviours”.[7] Programs have traditionally consisted of twice-weekly attendance as an outpatient in a group setting for 8-12 weeks.[8-9] Clinical trials show that outpatient pulmonary rehabilitation for COPD is highly effective, with Level 1 evidence for improved exercise capacity, reduced breathlessness and improved quality of life, regardless of disease severity.[10] Pulmonary rehabilitation also reduces acute exacerbations, decreases hospital days and reduces hospital admissions, with a concomitant reduction in health care costs.[11,12]

For peer review only - http://bmjopen.bmj.com/site/about/guidelines.xhtml

Despite compelling evidence for the benefits of pulmonary rehabilitation and unequivocal recommendations in COPD guidelines,[8] patient access to pulmonary rehabilitation remains limited worlwide.[14-19] Barriers preventing patients from accessing the benefits of pulmonary rehabilitation occur at a system level (shortage of programs) and individual level (poor physical mobility, distressing symptoms and inability to travel).[20-21] Between 8% and 50% of patients referred to a pulmonary rehabilitation program never attend, and of those who do start, between 10% and 32% do not complete a program.[20] A 2015 American Thoracic Society(ATS)/European Respiratory Society (ERS) policy statement called on the research community to increase access to pulmonary rehabilitation by establishing the efficacy of alternative models.[21]

A low cost model of delivering pulmonary rehabilitation directly into the home was developed (HomeBase) in Australia to improve access and uptake.[22] A previous Phase II randomised controlled equivalence trial[23] showed that HomeBase delivered equivalent clinical outcomes (exercise capacity, symptoms and quality of life) to traditional centre-based rehabilitation in COPD with similar costs and better completion rates (91% vs 49%). The clinical implementation of home-based pulmonary rehabilitation programs has the potential to improve health outcomes, reduce health care utilisation and minimise societal costs but should not be considered a replacement for traditional centre-based programs. Semi-structured interviews with participants who received home-based rehabilitation in the Phase II trial revealed that participants valued the convenience and flexibility of exercise training at home, and the reduced travel burden of a home-based program.[24]

For peer review only

For peer review only - http://bmjopen.bmj.com/site/about/guidelines.xhtml

However, 18% of individuals assessed for eligibility to that trial declined to participate because they wanted to be certain they would receive their preferred choice of a traditional centre-based program. [23]

A recent ATS workshop report identified that the emergence of alternative models of pulmonary rehabilitation pose many unanswered questions for clinical practice, which will be best addressed by prospective clinical implementation trials.[25] At a health service or program level it is likely that the best outcomes and optimal completion rates will be achieved by offering patients a choice of pulmonary rehabilitation locations. Our previous equivalence trial of home versus centre-based rehabilitation showed that patients who completed pulmonary rehabilitation, regardless of group allocation, were 56% less likely to be hospitalised in the following 12 months and had a longer time to their next hospital admission.[23] Offering the choice of home or centre-based pulmonary rehabilitation may increase program completion and therefore result in fewer hospitalisations and more patients with COPD achieving improvements in exercise capacity, breathlessness and quality of life. Until now, the offer of choice of pulmonary rehabilitation location versus traditional centre-based only has not been investigated in a clinical trial.

The aims of this study are to 1) compare hospitalisation in the 12 months following pulmonary rehabilitation for programs implementing a choice of location (home or centre) to those that offer centre-based only, 2) compare outcomes of programs implementing choice of location to those that offer centre-based only, and 3) compare health care costs in the 12 months following pulmonary rehabilitation for programs that offer choice of location to

For peer review only

For peer review only - http://bmjopen.bmj.com/site/about/guidelines.xhtml

those that offer centre-based only. We hypothesise that programs offering patient choice of pulmonary rehabilitation location will result in i) fewer hospitalisations in the 12 months following pulmonary rehabilitation as a result of increased program completion, compared to centre-based-only programs; ii) significantly better outcomes for exercise capacity, symptoms and health-related quality of life compared to centre-based-only programs; and lower health care costs as a result of reduced hospital admissions compared to centre-based-only programs.

For peer review only

For peer review only - http://bmjopen.bmj.com/site/about/guidelines.xhtml

Methods and analysis

Design

A two-arm cluster randomised, controlled, assessor-blinded trial will be conducted with 14 centre-based pulmonary rehabilitation services across Australia. The overall trial design is depicted in Figure 1. This trial protocol is reported in accordance with SPIRIT guidelines.[26] The trial has been prospectively registered at ClinicalTrials.gov, will be conducted in accordance with the principles of Good Clinical Practice and will be reported according to CONSORT[27] and StaRI[28] standards for cluster randomised controlled trials and implementation studies.

Clusters (Pulmonary rehabilitation programs)

Included pulmonary rehabilitation services must provide outpatient programs that admit at least 50 people with COPD each year and must be able to demonstrate established protocols for centre-based pulmonary rehabilitation according to national guidelines.[29] These sites are representative of the facilities where pulmonary rehabilitation is delivered in Australia, including community programs, and large and medium sized hospitals.

Randomisation

Eligible pulmonary rehabilitation services will be randomised 1:1 to either the intervention group (offering choice of home-based or centre-based pulmonary rehabilitation) or control group (continuing to offer only centre-based pulmonary rehabilitation) stratified by setting of existing centre-based pulmonary rehabilitation (hospital vs non hospital). Randomisation (sequence generation and allocation concealment) will be performed using a secure

For peer review only

online randomisation service managed by an independent agency. Block randomisation is being used to both balance cluster allocation within strata and to help ensure the methods (in particular the block size) can remain consistent throughout the trial (e.g. should clusters withdraw from the trial).

Blinding

Research staff: Site principal investigators at each cluster will be notified of their allocation by the research team. Clinicians within sites who are involved in delivery of the intervention will not be blinded to group allocation. However, research staff within clusters that conduct post-intervention assessments and enter questionnaire data into the trial database will be blinded to group allocation.

Participants: Participants will be blinded to group allocation. Patients will be informed that this study is examining the impact of pulmonary rehabilitation models on patient and health system outcomes. The specific nature of the models of interest, or the intervention group to which their site has been allocated will not be disclosed. As this is a study of behaviour (the impact of offering choice of pulmonary rehabilitation models), limited disclosure of the study aim regarding comparison of models is necessary. To truly understand and measure the impact of offering choice of program models, the offer of choice in the intervention groups will be made after participant consent for the trial has been obtained, so that the choice can be recorded.

For peer review only

For peer review only - http://bmjopen.bmj.com/site/about/guidelines.xhtml

Participants

To be eligible for inclusion at the 14 pulmonary rehabilitation services participants must i) have a diagnosis of COPD confirmed on spirometry; ii) be able to read, write and speak English; iii) be able to provide informed consent. Exclusion criteria include: i) attendance at pulmonary rehabilitation in the last 12 months; ii) comorbidities which preclude exercise training; iii) inability to provide informed consent.

Recruitment

Eligible participants will be identified through referrals to pulmonary rehabilitation programs at participating clusters. To ensure that referrals are maximised across all sites, respiratory and general physicians at their affiliated hospitals will receive three-monthly reminders to refer patients with COPD to pulmonary rehabilitation, consistent with current guidelines for best practice care (COPD-X).[30] Screening will be conducted by the site investigator, who is a staff member in the pulmonary rehabilitation program. Potential participants will be sent an information flier about the study prior to their initial pulmonary rehabilitation appointment. They will be invited to discuss the study further at their initial appointment, or by telephone.

Delivery of pulmonary rehabilitation

Participants in both groups will receive a copy of the 'Better Living with Exercise' booklet, produced by Lung Foundation Australia. The 'Better Living with Exercise' booklet assists people with COPD to work with their health professionals to optimise their exercise program, based on their individual health conditions, goals and interests. All participants will also receive details

For peer review only

to access an online version of the 'Better Living with COPD; A Patient Guide' on the Lung Foundation Australia website. The 'Better Living with COPD: A Patient Guide' (Third Edition)[31] was developed to support COPD patients to better understand and manage their condition. At the completion of the rehabilitation period participants will be offered the opportunity to join a supervised exercise maintenance program to promote ongoing exercise adherence in accordance with existing clinical practice at the trial site.

Control group: Pulmonary rehabilitation programs assigned to the control group will offer eligible participants the opportunity to participate in their usual centre-based pulmonary rehabilitation, delivered according to current best practice guidelines.[29] This will be an 8-week, twice weekly outpatient group-based supervised program, with individually prescribed exercise training and self-management education. At least 30 minutes of aerobic exercise training will be performed each session, plus resistance exercises using functional activities such as stair climbing and sit-to-stand practice, as well as free weights for the upper limbs. Participants will also be encouraged to exercise at home on 3 occasions each week and to record this in the Lung Foundation Australia 'Better Living with Exercise' resource diary. Programs may also include self-management training in structured (lecture-based) and unstructured disease management education on a group and/or 1:1 basis, in accordance with existing clinical practice at the trial site.

Intervention group: Pulmonary rehabilitation programs assigned to the intervention group will offer eligible participants the choice of participating in an 8 week program of either home-based or traditional centre-based pulmonary rehabilitation. Home-based rehabilitation will be delivered

For peer review only - http://bmjopen.bmj.com/site/about/guidelines.xhtml

according to our protocol (HomeBase), which has been published in detail previously.[22,23,32] Briefly, HomeBase includes the following components:

Home Visit: The program commences with one home visit by a physiotherapist experienced in pulmonary rehabilitation during which exercise goals are established, the first exercise session is supervised and aspects of the self-management program that cannot be adequately dealt with during subsequent telephone calls (e.g. review of inhaler technique), are covered.

Exercise training: Participants will follow an aerobic and strength training program, which will be supervised for the first session and unsupervised thereafter. For the aerobic training, a walking program will be prescribed with participants set a walking distance to be completed in a given time via a pedometer and on the basis of a baseline 6MWT (initial target intensity of 80% of the speed walked in the test). At least 30 minutes of aerobic exercise is recommended for each session, for at least 5 sessions per week. Strength training will utilise functional activities and equipment that are readily accessible in the home environment (e.g. sit-to-stand from a dining chair, water bottles for upper limb weights).

Home Exercise diary: The initial goals set for aerobic and resistance exercise and details of the supervised exercise session during the home visit will be recorded in a home exercise diary. Participants will be encouraged to document all subsequent program goals (exercise or health) and completed unsupervised exercise sessions, including duration and distance walked and the number and type of resistance exercises performed, in their diary each week. We have shown a significant relationship between diary-documented exercise sessions and device measured physical activity.[33] Participants will

For peer review only

also be asked to record what barriers may prevent them from achieving their goals or exercise and how they anticipate they may overcome these barriers. Prior to commencing each unsupervised exercise session, the home exercise diary prompts the participant to check whether they have any symptoms of moderate or severe exacerbation and to contact the pulmonary rehabilitation clinician at the site if symptomatic.

Weekly telephone calls: Following the home visit in week one, each participant will receive seven once-weekly telephone calls. All calls will delivered using a motivational interviewing approach[34] by a physiotherapist experienced in pulmonary rehabilitation and has undertaken undertake formal training in motivational interviewing for health care. Additional trial-based training and regular fidelity checks using the Motivational Interviewing Treatment Integrity Scale version 4.2.1[35] will also take place. During the telephone calls the physiotherapist will review the home exercise diary; progress the exercise prescription; review symptoms and facilitate self-management of exacerbations; and deliver self-management education via scripted telephonic modules. These structured telephone modules will be used to explore and build motivation for exercise participation.[22,23] The home exercise diary provides participants with a menu of topics related to self-care. Participants will be encouraged to select a topic they feel is relevant to them for discussion with the physiotherapist in their weekly call, providing opportunities for self-management education and goal setting. All participants will have a discussion on managing an acute exacerbation of their COPD and long-term exercise planning, consistent with the goals of centre-based pulmonary rehabilitation programs. Participants will have access to the 'Better

For peer review only - http://bmjopen.bmj.com/site/about/guidelines.xhtml

Living with COPD: A Patient Guide' and the C.O.P.E program (COPD Online Patient Education) to support topic discussions.

Data collection and follow up

Each participant will complete 8 weeks of rehabilitation and a follow-up of 12 months. Outcomes will be assessed at baseline, end of rehabilitation (after 8 weeks) and 12 months following the end of rehabilitation. Assessments will be undertaken at 8 weeks and 12 months irrespective of whether a participant has completed their pulmonary rehabilitation program. Participants will record any health care utilisation during the 12 months follow-up (visits to health professionals, hospitalisations) in a diary. Each participant will be telephoned monthly during the 12 months by site research staff to encourage diary completion and capture patient-reported information on health-care utilisation. Hospitalisation and use of other hospital services will be confirmed by medical record audit at 12 months, whilst 12-month medical and pharmaceutical resource use will be obtained from Services Australia using Pharmaceutical Benefits Scheme (PBS) and Medicare Benefits Schedule (MBS) data. Consent will be sought to use participants' PBS and MBS data at the time of consent to the study. The schedule for assessments and follow-up is provided in Table 1.

Outcomes

Primary Outcome

All-cause, non-elective hospitalisation over 12 months: The number of participants hospitalised at least once in the 12 months following pulmonary rehabilitation will be compared between groups. Avoidance of hospitalisation

For peer review only

For peer review only - http://bmjopen.bmj.com/site/about/guidelines.xhtml

is the research outcome that people with COPD value most highly and is of critical importance to the health system.[36] We have chosen all cause, non-elective hospitalisation due to the challenges in assessing whether an admission is COPD-related in a group where multimorbidity is ubiquitous (median 4 coexisting conditions in our previous trial[23]) and the potential for bias in adjudication of respiratory-related admissions significant. We have developed an effective method to collect these data in our previous trial[23] combining medical record audit with monthly telephone calls to participants, to ensure comprehensive coverage of health care utilisation.

Secondary outcomes

Program completion: The number of participants who complete their allocated pulmonary rehabilitation program will be compared between groups at 8 weeks. Completion is defined as undertaking 70% of planned sessions in accordance with recent recommednations.[25]

6-minute walk distance (6MWD): The 6MWD is a validated measure of functional exercise capacity for COPD.[37] The 6MWD predicts both future hospitalisation[38] and survival.[39] It is responsive to change following pulmonary rehabilitation[10] and is an outcome that matters to patients.[40] The test will be performed in accordance with international standards,[37] including two tests at each time point, with the better (i.e. longest distance) 6MWD recorded.

Chronic Respiratory Questionnaire (CRQ): The CRQ is a disease-specific health-related quality of life measure with domains of dyspnoea, fatigue, mastery and emotional function. It is a patient-centred outcome that is responsive to change following pulmonary rehabilitation.[10] The self-reported version of the CRQ[41] will be used.

For peer review only

EQ-5D-5L: This validated generic quality of life measure[42] is used to estimate health benefits in terms of quality-adjusted life-years (QALYs),[43]and is recommended for economic analyses.

Dyspnoea-12: This a global measure of breathlessness severity that captures both the physical and affective components of dyspnoea. It has excellent internal consistency, validity and reliability in COPD.[44]

Physical activity participation: This is an important measure of behaviour change following pulmonary rehabilitation. We will measure time spent in moderate to vigorous physical activity and sedentary time, using the ActiGraph GT3X, a waist-worn, tri-axial accelerometer that has been validated in people with COPD.[45] Seven days of monitoring will be performed to accurately capture all activity intensities.[46]

Health care utilisation across 12 months: Healthcare utilisation will be recorded monthly in participant diaries and telephone record sheets kept by site research staff. We will confirm health care utilisation from hospital records, MBS and PBS data.

Sample size

The primary outcome is hospitalisation in the 12 months following pulmonary rehabilitation. In our published Phase II trial,[23] 57% of the centre-based group were free from hospitalisation at 12 months, whereas 78% of rehabilitation completers were free from hospitalisation (regardless of group). Completion was 91% for HomeBase, and 49% for centre-based.[23] The novel aspect of the current study is that intervention participants will be offered a choice of models. Based on our experience recruiting for HomeBase, we expect that 75% of individuals may select a home program of whom 91% will complete,

For peer review only - http://bmjopen.bmj.com/site/about/guidelines.xhtml

387 and 25% will select centre-based of whom 49% will complete. We thus
388 anticipate a completion rate of 80% in the intervention (choice) group.
389 At 80% completion, our data indicate that 72% of the intervention group would
390 remain free from admission at 12 months, compared to the observed rate of
391 57% in centre-based (control) participants.[23] We thus expect 15% reduction in
392 hospitalisations in the intervention group (choice of home-based or centre-
393 based rehabilitation) compared to control (centre-based rehabilitation only).
394 To detect a difference in remaining free from hospitalisation between
395 intervention and control groups of 15% (72% vs 57%) with 80% power and a
396 2-sided 0.05 significance level, 312 participants (156 in each group) are
397 required. Adjusting for clustering by program (intra-cluster correlation
398 coefficient of 0.01, based on our published 2-site trial, and 35 participants per
399 cluster), the total required sample is 418. Our previous study had 10% loss to
400 follow-up, so to allow for this we will recruit a total of 490 participants from 14
401 pulmonary rehabilitation programs.

402 **Statistical analysis**

403 Generalised linear mixed models will be used to analyse the primary outcome
404 of hospitalisation, and linear mixed models will be used for continuous
405 outcomes. These will account for correlation between participants within a
406 cluster. Analyses will be based on intention-to-treat and the level of
407 significance will be set at $p < 0.05$.

408 **Economic Evaluation**

409 Economic evaluation will follow the protocol developed for our Phase II
410 trial.[22,23]

For peer review only - http://bmjopen.bmj.com/site/about/guidelines.xhtml

Cost comparisons: Cost comparisons will include comparison of per person costs, including direct (health system) and indirect (personal) health care costs for the two groups. Direct costs will include staff time, consumables, communications and overheads. Intervention costs will include staff inputs by duration, type and resource use (including troubleshooting and support) and equipment. Personal costs will include transportation, travel time and impact of the intervention on the economic activities of other household members. Health system costs will include visits to the general practitioner, specialist or emergency department; use of chronic disease services; and hospitalisation.

Cost-effectiveness analysis: An incremental cost-effectiveness analysis will be undertaken to compare differences in costs between the two groups with differences in quality adjusted life years (QALYs) and number of hospitalisations in the 12-month follow-up period. For the former, a utility index (UI) will be calculated from the EQ-5D-5L by applying a 'social tariff', which then enables estimation of health benefits in terms of QALYs. The conversion to QALYs will be based on the assumption that for health states measured at successive points of time, the duration of each health state is exactly one half of the time interval between the two measurements.[47] For the latter, the indicator of cost effectiveness can be interpreted as the incremental cost of avoiding a hospitalisation.

Process evaluation

A process evaluation, informed by the Medical Research Council Guidance on Process Evaluations of complex interventions,[48] is also planned for this trial. Offering a choice of program models represents a departure from traditional care. Thus, understanding both clinician and patient experiences of this new

For peer review only

For peer review only - http://bmjopen.bmj.com/site/about/guidelines.xhtml

approach is critical to guide scale-up and future implementation. A detailed protocol for the process evaluation will be published separately. Briefly, the process evaluation will undertake a theory-based approach using the Theoretical Domains Framework (TDF).[49] The deductive coding using the TDF will allow the team to understand clinician or patient behaviours that act as barriers or facilitators to implementation of HomeBase, and to develop a compendium of implementation strategies for future scale-up. Implementation will be further explored using the RE-AIM (Reach, Effectiveness, Adoption, Implementation, and Maintenance) framework[50] with collection of both quantitative and qualitative data throughout the trial to provide insights into translation and behaviour change success in intervention clusters.

Ethics and dissemination

This protocol (version 2, 16 August 2020) has received approval from Alfred Hospital Human Research Ethics Committee (379/19). Local research ethics committee approvals have also been obtained at sites where the National Mutual Acceptance scheme does not apply. Each site is also required to obtain local governance approval prior to commencement of data collection. Site investigators will be informed by the trial coordinator of any necessary modifications to the protocol and amendments to local ethics and/or governance approval. Potential participants at each site may be in a dependent relationship with pulmonary rehabilitation staff. For this reason pulmonary rehabilitation staff will only provide initial information about the trial to a potential participant, either at the time of initial assessment or over the telephone. If the potential participant is interested in receiving further detailed information, or providing informed consent, a researcher who is not in a

For peer review only

For peer review only - http://bmjopen.bmj.com/site/about/guidelines.xhtml

dependent relationship with the potential participant will undertake this. The trial findings will be published in peer-reviewed journals and submitted to national and international conferences for presentation. The authorship of any publications will adhere to the guidelines established by the International Committee of Medical Journal Editors. We will disseminate results to patients with COPD across Australia through Lung Foundation Australia's e-newsletter and website, and to health professionals through Thoracic Society of Australia and New Zealand's e-news, scientific conferences and webinar program.

For peer review only

Safety monitoring and reporting

All sites will maintain an adverse event log for the trial period. An adverse event in the trial is being defined as any unfavourable, unintended diagnosis, symptom, sign (including an abnormal laboratory finding), syndrome, or disease that occurs during the trial, having been absent at baseline, or if present at baseline, appears to have worsened. A serious adverse event is defined as any adverse event leading to i) death; ii) serious deterioration in health resulting in a life-threatening illness or injury, or permanent impairment of a body structure or a body function; iii) hospitalisation or prolongation of existing hospitalisation; iv) medical or surgical intervention to prevent life-threatening illness; v) foetal distress, foetal death or a congenital abnormality. Sites will inform the trial coordinator of any adverse event that necessitates a change to the protocol or participant information and consent form, is unexpected, or any serious adverse events. The trial coordinator will ensure the data and safety monitoring board (DSMB), relevant research ethics committees or governance offices are informed of any serious adverse events where necessary. The DSMB, consisting of a respiratory physician, two

For peer review only - http://bmjopen.bmj.com/site/about/guidelines.xhtml

physiotherapists and a statistician, will make the final decision on the relatedness of any adverse events to the trial interventions or procedures. Unless otherwise determined by trial events, the DSMB will convene bi-annually.

Data management

Hard copy original data collection forms will be stored in a locked filing cabinet within a locked office at each trial site. Electronic data will be stored centrally in a purpose-built on-line database (www.adeptrs.com), with encryption and password protection. No identifying information will be stored in the online database. Electronic data for all sites will be accessible by the coordinating principal investigator and the trial coordinator. Site specific investigators will only have access to data relating to their individual site.

Patient and public involvement

The experiences of HomeBase participants in our previous Phase II trial[24] were critical to formulating the current proposal. Hospitalisation has been chosen as the primary outcome of this trial because it is the research outcome that is most meaningful for patients with COPD.[36] Two patients with COPD will sit on the trial steering committee. All patient-facing trial resources (eg fliers, Participant Information and Consent Forms, diaries) have undergone review by patients with COPD via Lung Foundation Australia's COPD Patient Advisory Group.

Trial status

Trial recruitment began in March 2021 and is ongoing.

For peer review only

For peer review only - http://bmjopen.bmj.com/site/about/guidelines.xhtml

Discussion

This research will be the first randomised controlled trial to determine the clinical benefits and health care costs of offering patients a choice of two pulmonary rehabilitation locations (home- or centre-based) compared to offering only traditional centre-based models. Until recently, there has been very little “choice” for patients referred to pulmonary rehabilitation; the delivery model of centre-based pulmonary rehabilitation has largely been a “one size fits all” that is inconsistent with the personalised approach of modern medicine.[25]

The adoption of alternative models for pulmonary rehabilitation will rely on demonstration of comparable or better clinical outcomes compared with those of traditional pulmonary rehabilitation programs, which can be delivered in a cost-effective manner.[51] A novel aspect of this trial is that our intervention will offer patients with COPD the choice of two rehabilitation models that are underpinned by strong efficacy data, an approach that provides patients with increased opportunities to attain the benefits of pulmonary rehabilitation. Our previous economic analysis[52] suggested that completion of pulmonary rehabilitation is an independent predictor of lower health care costs in COPD.

Based on head-to-head trial evidence of home versus outpatient-based pulmonary rehabilitation, the 2017 Australian Pulmonary Rehabilitation guidelines recommended home programs as an alternative to hospital-based programs.[29] The offer of a home-based program has long been a limited option in pulmonary rehabilitation services.[16] The coronavirus disease (COVID-19) pandemic had led to many pulmonary rehabilitation services

For peer review only - http://bmjopen.bmj.com/site/about/guidelines.xhtml

rapidly transitioning to home-based models but there remains little information about how best to implement these models in a clinical setting.[25] Home-based programs have the potential to address many of the patient-related and system-related barriers to attending traditional centre-based pulmonary rehabilitation models in COPD including improvements to access (e.g. greater scope in program delivery) and uptake (allowing patient preference for home-based care, reducing barriers related to travel and disability). Targeted implementation is required to translate the clinical efficacy of home-based programs to important health outcomes in routine practice. Implementation of home-based programs alongside the traditional outpatient centre-based models may allow evidence-based pulmonary rehabilitation to be more accessible to patients. A process evaluation will be conducted alongside the cluster randomised controlled trial in order to understand barriers and facilitators to offering choice of pulmonary rehabilitation models at trial sites, and to provide data that will guide future implementation.

This trial will provide the evidence needed to underpin future guidelines and policy decisions for pulmonary rehabilitation. If successful, the findings of this trial can be readily implemented into clinical practice worldwide. Ultimately, the findings will inform a key change in the future of pulmonary rehabilitation services, where delivery includes more choices for patients and opportunities for greater personalisation of programs.

For peer review only

References

1. Global Strategy for the Diagnosis, Management, and Prevention of COPD 2021 report, Global Initiative for Chronic Obstructive Lung Disease (GOLD) [Accessed 4 June 2021]. Available from: https://goldcopd.org/2021-gold-reports/
2. Hanania NA, O'Donnell DE. Activity-related dyspnea in chronic obstructive pulmonary disease: physical and psychological consequences, unmet needs, and future directions. I*nt J Chron Obstruct Pulmon Dis* 2019;14:1127-1138.
3. Spruit MA, Burtin C, De Boever P, Langer D, Vogiatzis I, Wouters EF, Franssen FM. COPD and exercise: does it make a difference? *Breathe* 2016;12(2):38-49.
4. Australian Institute of Health and Welfare 2017. Web Update: Potentially preventable hospitalisations in 2015-16 [Accessed 28 December 2018]. Available from: https://www.myhealthycommunities.gov.au/our-reports/potentially-preventable-hospitalisations-update/july-2017 =
5. Miravitlles M, Garcia-Polo C, Domenech A, et al. Clinical outcomes and cost-analysis of exacerbations in chronic obstructive pulmonary disease. *Lung* 2013; 191:523-530
6. Wouters EF. Economic analysis of the Confronting COPD survey: an overview of results. *Respir Med* 2003; 97:S3-14
7. Spruit M, Singh S, Garvey C, *et al*. An official American Thoracic Society/European Respiratory Society statement: key concepts and advances in pulmonary rehabilitation. *Am J Resp Crit Care Med* 2013;188:13-64.
8. Holland AE, Cox NS, Houchen-Wolloff L, Rochester CL, Garvey C, ZuWallack R, Nici L, Limberg T, Lareau SC, Yawn BP, Galwicki M, Troosters T, Steiner M, Casaburi R, Clini E, Goldstein RS, Singh SJ. Defining Modern Pulmonary Rehabilitation. An Official American Thoracic Society Workshop Report. *Ann Am Thorac Soc* 2021;18(5):e12-e29.
9. Spruit MA, Pitta F, Garvey C, ZuWallack RL, Roberts CM, Collins EG, et al.; ERS Rehabilitation and Chronic Care, and Physiotherapists Scientific Groups; American Association of Cardiovascular and Pulmonary Rehabilitation; ATS Pulmonary Rehabilitation Assembly and the ERS COPD Audit team. Differences in content and organisational aspects of pulmonary rehabilitation programmes. *Eur Respir J* 2014;43:1326–1337.
10. McCarthy B, Casey D, Devane D, *et al*. Pulmonary rehabilitation for chronic obstructive pulmonary disease. *Cochrane Database Syst Rev* 2015;23
11. Griffiths TL, Phillips CJ, Davies S, Burr ML, Campbell IA. Cost effectiveness of an outpatient multidisciplinary pulmonary rehabilitation programme. *Thorax* 2001; 56: 779-84

For peer review only

For peer review only - http://bmjopen.bmj.com/site/about/guidelines.xhtml

12. Guell R, Casan P, Belda J, Sangenis M, Morante F, Guyatt GH, Sanchis J. Long-term effects of outpatient rehabilitation of COPD: A randomized trial. *Chest* 2000; 117: 976-83.

13. Moore E, Palmer T, Newson R, Majeed A, Quint JK, Soljak MA. Pulmonary Rehabilitation as a Mechanism to Reduce Hospitalizations for Acute Exacerbations of COPD: A Systematic Review and Meta-Analysis. *Chest* 2016;150(4):837-859.

14. Brooks D, Sottana R, Bell B, Hanna M, Laframboise L, Selvanayagarajah S, Goldstein R. Characterization of pulmonary rehabilitation programs in Canada in 2005. Can Respir J 2007;14(2):87-92

15. Camp PG, Hernandez P, Bourbeau J, Kirkham A, Debigare R, Stickland MK, Goodridge D, Marciniuk DD, Road JD, Bhutani M, Dechman G. Pulmonary rehabilitation in Canada: A report from the Canadian Thoracic Society COPD Clinical Assembly. *Can Respir J* 2015;22(3):147-52.

16. Marks G, Reddel H, Guevara-Rattray E, Poulos L, Ampon R. Monitoring pulmonary rehabilitation and long-term oxygen therapy for people with chronic obstructive pulmonary disease (COPD) in Australia: a discussion paper. Australian Institute of Health and Welfare, Canberra, 2013 [Accessed 4 June 2021] Available from: https://www.aihw.gov.au/reports/chronic-respiratory-conditions/monitoring-pulmonary-rehabilitation-and-long-term/contents/table-of-contents.

17. Nishi SP, Zhang W, Kuo YF, Sharma G. Pulmonary Rehabilitation Utilization in Older Adults With Chronic Obstructive Pulmonary Disease, 2003 to 2012. *J Cardiopulm Rehabil Prev* 2016;36(5):375-82.

18. Steiner M, Holzhauer-Barrie J, Lowe D, Searle L, Skipper E, Welham S, Roberts CM. Pulmonary Rehabilitation: Time to breathe better. National Chronic Obstructive Pulmonary Disease (COPD) Audit Programme: Resources and organisation of Pulmonary Rehabilitation services in England and Wales 2015. National organisational audit report. Royal College of Physicians, London, 2015 [Accessed 4 June 2021]. Available from: https://www.rcplondon.ac.uk/projects/outputs/pulmonary-rehabilitation-time-breathe-better

19. Yohannes AM, Connolly MJ. Pulmonary rehabilitation programmes in the UK: a national representative survey. *Clin Rehabil* 2004;18(4):444-9.

20. Keating A, Lee A, Holland AE. What prevents people with chronic obstructive pulmonary disease from attending pulmonary rehabilitation? A systematic review. *Chron Respir Dis* 2011; 8: 89-99.

21. Rochester CL, Vogiatzis I, Holland AE, Lareau SC, Marciniuk DD, Puhan MA, Spruit MA, Masefield S, Casaburi R, Clini EM, Crouch R, Garcia-Aymerich J, Garvey C, Goldstein RS, Hill K, Morgan M, Nici L, Pitta F, Ries AL, Singh SJ, Troosters T, Wijkstra PJ, Yawn BP, ZuWallack RL; ATS/ERS Task Force on Policy in Pulmonary Rehabilitation. An Official American Thoracic Society/European Respiratory Society Policy Statement: Enhancing Implementation, Use, and Delivery of Pulmonary Rehabilitation. *Am J Respir Crit Care Med* 2015;192(11):1373-86.

For peer review only - http://bmjopen.bmj.com/site/about/guidelines.xhtml

22. Holland AE, Mahal A, Hill CJ, Lee AL, Burge AT, Moore R, Nicolson C, O'Halloran P, Cox NS, Lahham A, Ndongo R, Bell E, McDonald CF. Benefits and costs of home-based pulmonary rehabilitation in chronic obstructive pulmonary disease - a multi-centre randomised controlled equivalence trial. *BMC Pulm Med* 2013;13:57.

23. Holland AE, Mahal A, Hill CJ, Lee AL, Burge AT, Cox NS, Moore R, Nicolson C, O'Halloran P, Lahham A, Gillies R, McDonald CF. Home-based rehabilitation for COPD using minimal resources: a randomised, controlled equivalence trial. *Thorax* 2017;72(1):57-65.

24. Lahham A, McDonald CF, Mahal A, Lee AL, Hill CJ, Burge AT, Cox NS, Moore R, Nicolson C, O'Halloran P, Gillies R, Holland AE. Home-based pulmonary rehabilitation for people with COPD: A qualitative study reporting the patient perspective. *Chron Respir Dis* 2018;15(2):123-130.

25. Holland AE, Cox NS, Houchen-Wolloff L, Rochester CL, Garvey C, ZuWallack R, Nici L, Limberg T, Lareau SC, Yawn BP, Galwicki M, Troosters T, Steiner M, Casaburi R, Clini E, Goldstein RS, Singh SJ. Defining Modern Pulmonary Rehabilitation. An Official American Thoracic Society Workshop Report. *Ann Am Thorac Soc* 2021;18(5):e12-e29.

26. Chan AW, Tetzlaff JM, Gøtzsche PC, Altman DG, Mann H, Berlin JA, Dickersin K, Hróbjartsson A, Schulz KF, Parulekar WR, Krleza-Jeric K, Laupacis A, Moher D. SPIRIT 2013 explanation and elaboration: guidance for protocols of clinical trials. *BMJ* 2013;346:e7586.

27. Campbell M K, Piaggio G, Elbourne D R, Altman D G. Consort 2010 statement: extension to cluster randomised trials *BMJ* 2012;345 :e5661.

28. Pinnock H, Barwick M, Carpenter CR, Eldridge S, Grandes G, Griffiths CJ, Rycroft-Malone J, Meissner P, Murray E, Patel A, Sheikh A, Taylor SJ, Sta RIG. Standards for Reporting Implementation Studies (StaRI) Statement. *BMJ* 2017; 356: i6795

29. Alison JA, McKeough ZJ, Johnston K, McNamara RJ, Spencer LM, Jenkins SC, Hill CJ, McDonald VM, Frith P, Cafarella P, Brooke M, Cameron-Tucker HL, Candy S, Cecins N, Chan AS, Dale MT, Dowman LM, Granger C, Halloran S, Jung P, Lee AL, Leung R, Matulick T, Osadnik C, Roberts M, Walsh J, Wootton S, Holland AE. Australian and New Zealand Pulmonary Rehabilitation Guidelines. *Respirology* 2017; 22 (4):800–819.

30. Yang IA, George J, McDonald CF, McDonald V, O'Brien M, Smith B, Zwar N, Dabscheck E. The COPD-X Plan: Australian and New Zealand Guidelines for the management of Chronic Obstructive Pulmonary Disease 2021. Version 2.63, February 2021.

31. Queensland Health Statewide Respiratory Clinical Network and Lung Foundation Australia. Better Living with Chronic Obstructive Pulmonary Disease A Patient Guide. 3rd ed, Queensland, 2016.

32. HomeBase Rehab [Accessed 4 June 2021]. Available from: https://homebaserehab.net/

For peer review only - http://bmjopen.bmj.com/site/about/guidelines.xhtml

33. Lahham A, McDonald CF, Mahal A, Lee AL, Hill CJ, Burge AT, Cox NS, Moore R, Nicolson C, O'Halloran P, Gillies R, Holland AE. Acceptability and validity of a home exercise diary used in home-based pulmonary rehabilitation: A secondary analysis of a randomised controlled trial. *Clin Respir J* 2018; 12: 2057-64.

34. Miller WR, Rollnick S R. Motivational interviewing: Helping people change. 3rd ed. NY: Guildford Press 2013.

35. Moyers TB, Rowell LN, Manuel JK, et al. The Motivational Interviewing Treatment Integrity Code (MITI 4): Rationale, Preliminary Reliability and Validity. J Subst Abuse Treat 2016;65:36-42. doi: 10.1016/j.jsat.2016.01.001 [published Online First: 2016/02/15]

36. Zhang Y, Morgan RL, Alonso-Coello P, Wiercioch W, Bala MM, Jaeschke RR, Styczen K, Pardo-Hernandez H, Selva A, Ara Begum H, Morgano GP, Waligora M, Agarwal A, Ventresca M, Strzebonska K, Wasylewski MT, Blanco-Silvente L, Kerth JL, Wang M, Zhang Y, Narsingam S, Fei Y, Guyatt G, Schunemann HJ. A systematic review of how patients value COPD outcomes. *Eur Respir J* 2018; 52.

37. Holland AE, Spruit MA, Troosters T, Puhan MA, Pepin V, Saey D, McCormack MC, Carlin BW, Sciurba FC, Pitta F, Wanger J, MacIntyre N, Kaminsky DA, Culver BH, Revill SM, Hernandes NA, Andrianopoulos V, Camillo CA, Mitchell KE, Lee AL, Hill CJ, Singh SJ. An official European Respiratory Society/American Thoracic Society Technical Standard: field walking tests in chronic respiratory disease. *Eur Respir J* 2014; 44: 1428-46.

38. Spruit MA, Polkey MI, Celli B, Edwards LD, Watkins ML, Pinto-Plata V, Vestbo J, Calverley PM, Tal-Singer R, Agusti A, Coxson HO, Lomas DA, Macnee W, Rennard S, Silverman EK, Crim CC, Yates J, Wouters EF. Predicting Outcomes from 6-Minute Walk Distance in Chronic Obstructive Pulmonary Disease. *J Am Med Dir Assoc* 2012; 13: 291-7.

39. Cote CG, Pinto-Plata V, Kasprzyk K, Dordelly LJ, Celli BR. The 6-min walk distance, peak oxygen uptake, and mortality in COPD. *Chest* 2007; 132: 1778-85.

40. Holland AE, Hill CJ, Rasekaba T, Lee A, Naughton MT, McDonald CF. Updating the minimal important difference for six-minute walk distance in patients with chronic obstructive pulmonary disease. *Arch Phys Med Rehabil* 2010; 91: 221-5.

41. Williams J, Singh S, Sewell L, Guyatt GH, Morgan MDL. Development of a self-reported Chronic Respiratory Questionnaire (CRQ-SR). *Thorax* 2001; 56: 954-959.

42. Nolan CM, Longworth L, Lord J, Canavan JL, Jones SE, Kon SS, Man WD. The EQ-5D-5L health status questionnaire in COPD: validity, responsiveness and minimum important difference. *Thorax* 2016;71(6):493-500.

43. Herdman M, Gudex C, Lloyd A, Janssen M, Kind P, Parkin D, Bonsel G, Badia X. Development and preliminary testing of the new five-level version of EQ-5D (EQ-5D-5L). *Qual Life Res* 2011;20(10):1727-36.

For peer review only

For peer review only - http://bmjopen.bmj.com/site/about/guidelines.xhtml

44. Yorke J, Moosavi SH, Shuldham C, Jones PW. Quantification of dyspnoea using descriptors: development and initial testing of the Dyspnoea-12. *Thorax* 2010; 65: 21-6.

45. Van Remoortel H, Raste Y, Louvaris Z, Van Remoortel, H., Raste, Y., Louvaris, Z., Giavedoni, S., Burtin, C., Langer, D., Wilson, F., Rabinovich, R., Vogiatzis, I., Hopkinson, N. S., Troosters, T., & PROactive consortium. Validity of six activity monitors in chronic obstructive pulmonary disease: a comparison with indirect calorimetry. *PLoS One* 2012; **7**: e39198.

46. Byrom B, Rowe DA. Measuring free-living physical activity in COPD patients: Deriving methodology standards for clinical trials through a review of research studies. *Contemp Clin Trials* 2016;47: 172-184.

47. Sinnott PL, Joyce VR, Barnett PG. Preference Measurement in Economic Analysis. Guidebook. Health Economics Resource Center, Menlo Park CA. VA Palo Alto, 2007.

48. Moore GF, Audrey S, Barker M, et al. Process evaluation of complex interventions: medical Research Council guidance. *BMJ* 2015;350:h1258.

49. Cane J, O'Connor D, Michie S. Validation of the theoretical domains framework for use in behaviour change and implementation research. *Implementation Science : IS*. 2012; **7**: 37.

50. Glasgow RE, Vogt TM, Boles SM. Evaluating the public health impact of health promotion interventions: the RE-AIM framework. *Am J Public Health* 1999;89:1322–7.

51. Rochester CL, Vogiatzis I, Holland AE, Lareau SC, Marciniuk DD, Puhan MA, Spruit MA, Masefield S, Casaburi R, Clini EM, Crouch R, Garcia-Aymerich J, Garvey C, Goldstein RS, Hill K, Morgan M, Nici L, Pitta F, Ries AL, Singh SJ, Troosters T, Wijkstra PJ, Yawn BP, ZuWallack RL; ATS/ERS Task Force on Policy in Pulmonary Rehabilitation. An Official American Thoracic Society/European Respiratory Society Policy Statement: Enhancing Implementation, Use, and Delivery of Pulmonary Rehabilitation. *Am J Respir Crit Care Med* 2015;192(11):1373-86.

52. Burge AT, Holland AE, McDonald CF, Abramson MJ, Hill CJ, Lee AL, Cox NS, Moore R, Nicolson C, O'Halloran P, Lahham A, Gillies R, Mahal A. Home-based pulmonary rehabilitation for COPD using minimal resources: An economic analysis. *Respirology* 2020;25(2):183-190.

For peer review only

For peer review only - http://bmjopen.bmj.com/site/about/guidelines.xhtml

Table 1: Schedule of trial follow-up and procedures

Assessment/ Procedure	Baseline	End rehabilitation program	Monthly phone calls	12 months post rehabilitation program
**Informed consent**	X			
**Demographic information**	X			
**Program completion**		X		
**6-minute walk test**	X	X		X
**Dyspnoea-12**	X	X		X
**Health related quality of life (EQ-5D-5L and CRQ)**	X	X		X
**Physical activity participation**	X	X		X
**Healthcare utilisation**			X	X
**Economic evaluation**			X	X

CRQ – chronic respiratory questionnaire.

Figure 1: Design of the HomeBase2 trial.

For peer review only

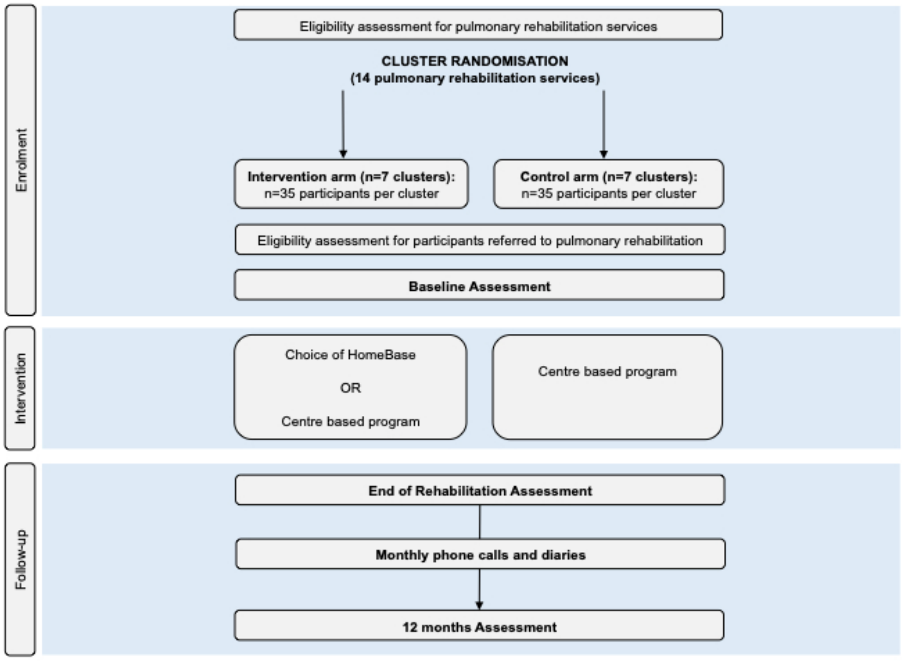

Design of the HomeBase2 trial

254x190mm (72 x 72 DPI)

SPIRIT

STANDARD PROTOCOL ITEMS: RECOMMENDATIONS FOR INTERVENTIONAL TRIALS

SPIRIT 2013 Checklist: Recommended items to address in a clinical trial protocol and related documents*

Section/item	Item No	Description	Addressed on page number
**Administrative information**			
Title	1	Descriptive title identifying the study design, population, interventions, and, if applicable, trial acronym	_____1________
Trial registration	2a	Trial identifier and registry name. If not yet registered, name of intended registry	_____2________
	2b	All items from the World Health Organization Trial Registration Data Set	Trial Register____
Protocol version	3	Date and version identifier	_____20_______
Funding	4	Sources and types of financial, material, and other support	_______1________
Roles and responsibilities	5a	Names, affiliations, and roles of protocol contributors	_______1______
	5b	Name and contact information for the trial sponsor	_______1________
	5c	Role of study sponsor and funders, if any, in study design; collection, management, analysis, and interpretation of data; writing of the report; and the decision to submit the report for publication, including whether they will have ultimate authority over any of these activities	_______1______
	5d	Composition, roles, and responsibilities of the coordinating centre, steering committee, endpoint adjudication committee, data management team, and other individuals or groups overseeing the trial, if applicable (see Item 21a for data monitoring committee)	_______20-22____

For peer review only

**Introduction**			
Background and rationale	6a	Description of research question and justification for undertaking the trial, including summary of relevant studies (published and unpublished) examining benefits and harms for each intervention	_______4-7______
	6b	Explanation for choice of comparators	________6_______
Objectives	7	Specific objectives or hypotheses	_______6-7______
Trial design	8	Description of trial design including type of trial (eg, parallel group, crossover, factorial, single group), allocation ratio, and framework (eg, superiority, equivalence, noninferiority, exploratory)	________8_______
**Methods: Participants, interventions, and outcomes**			
Study setting	9	Description of study settings (eg, community clinic, academic hospital) and list of countries where data will be collected. Reference to where list of study sites can be obtained	________8_______
Eligibility criteria	10	Inclusion and exclusion criteria for participants. If applicable, eligibility criteria for study centres and individuals who will perform the interventions (eg, surgeons, psychotherapists)	________8 & 10___
Interventions	11a	Interventions for each group with sufficient detail to allow replication, including how and when they will be administered	_______10-14____
	11b	Criteria for discontinuing or modifying allocated interventions for a given trial participant (eg, drug dose change in response to harms, participant request, or improving/worsening disease)	_________N/A___
	11c	Strategies to improve adherence to intervention protocols, and any procedures for monitoring adherence (eg, drug tablet return, laboratory tests)	_____15________
	11d	Relevant concomitant care and interventions that are permitted or prohibited during the trial	_______11-12____
Outcomes	12	Primary, secondary, and other outcomes, including the specific measurement variable (eg, systolic blood pressure), analysis metric (eg, change from baseline, final value, time to event), method of aggregation (eg, median, proportion), and time point for each outcome. Explanation of the clinical relevance of chosen efficacy and harm outcomes is strongly recommended	_______14-16____
Participant timeline	13	Time schedule of enrolment, interventions (including any run-ins and washouts), assessments, and visits for participants. A schematic diagram is highly recommended (see Figure)	14, Table 1 & Figure 1

For peer review only - http://bmjopen.bmj.com/site/about/guidelines.xhtml

Sample size	14	Estimated number of participants needed to achieve study objectives and how it was determined, including clinical and statistical assumptions supporting any sample size calculations	_____16-17____
Recruitment	15	Strategies for achieving adequate participant enrolment to reach target sample size	_______10______

Methods: Assignment of interventions (for controlled trials)

Allocation:

Sequence generation	16a	Method of generating the allocation sequence (eg, computer-generated random numbers), and list of any factors for stratification. To reduce predictability of a random sequence, details of any planned restriction (eg, blocking) should be provided in a separate document that is unavailable to those who enrol participants or assign interventions	_____8-9______
Allocation concealment mechanism	16b	Mechanism of implementing the allocation sequence (eg, central telephone; sequentially numbered, opaque, sealed envelopes), describing any steps to conceal the sequence until interventions are assigned	____8-9_______
Implementation	16c	Who will generate the allocation sequence, who will enrol participants, and who will assign participants to interventions	_____8-9______
Blinding (masking)	17a	Who will be blinded after assignment to interventions (eg, trial participants, care providers, outcome assessors, data analysts), and how	_____9________
	17b	If blinded, circumstances under which unblinding is permissible, and procedure for revealing a participant's allocated intervention during the trial	______N/A______ _

Methods: Data collection, management, and analysis

Data collection methods	18a	Plans for assessment and collection of outcome, baseline, and other trial data, including any related processes to promote data quality (eg, duplicate measurements, training of assessors) and a description of study instruments (eg, questionnaires, laboratory tests) along with their reliability and validity, if known. Reference to where data collection forms can be found, if not in the protocol	_____14-16_____
	18b	Plans to promote participant retention and complete follow-up, including list of any outcome data to be collected for participants who discontinue or deviate from intervention protocols	______14______

Data management	19	Plans for data entry, coding, security, and storage, including any related processes to promote data quality (eg, double data entry; range checks for data values). Reference to where details of data management procedures can be found, if not in the protocol	______21_______
Statistical methods	20a	Statistical methods for analysing primary and secondary outcomes. Reference to where other details of the statistical analysis plan can be found, if not in the protocol	______17-18____
	20b	Methods for any additional analyses (eg, subgroup and adjusted analyses)	______17_______
	20c	Definition of analysis population relating to protocol non-adherence (eg, as randomised analysis), and any statistical methods to handle missing data (eg, multiple imputation)	________17______
**Methods: Monitoring**			
Data monitoring	21a	Composition of data monitoring committee (DMC); summary of its role and reporting structure; statement of whether it is independent from the sponsor and competing interests; and reference to where further details about its charter can be found, if not in the protocol. Alternatively, an explanation of why a DMC is not needed	________20-21___
	21b	Description of any interim analyses and stopping guidelines, including who will have access to these interim results and make the final decision to terminate the trial	______N/A_____
Harms	22	Plans for collecting, assessing, reporting, and managing solicited and spontaneously reported adverse events and other unintended effects of trial interventions or trial conduct	______20-21____
Auditing	23	Frequency and procedures for auditing trial conduct, if any, and whether the process will be independent from investigators and the sponsor	______N/A______ _
**Ethics and dissemination**			
Research ethics approval	24	Plans for seeking research ethics committee/institutional review board (REC/IRB) approval	_____20________
Protocol amendments	25	Plans for communicating important protocol modifications (eg, changes to eligibility criteria, outcomes, analyses) to relevant parties (eg, investigators, REC/IRBs, trial participants, trial registries, journals, regulators)	_____19________

For peer review only - http://bmjopen.bmj.com/site/about/guidelines.xhtml

Consent or assent	26a	Who will obtain informed consent or assent from potential trial participants or authorised surrogates, and how (see Item 32)	____19_________
	26b	Additional consent provisions for collection and use of participant data and biological specimens in ancillary studies, if applicable	_______N/A______
Confidentiality	27	How personal information about potential and enrolled participants will be collected, shared, and maintained in order to protect confidentiality before, during, and after the trial	_______21_______
Declaration of interests	28	Financial and other competing interests for principal investigators for the overall trial and each study site	________1______
Access to data	29	Statement of who will have access to the final trial dataset, and disclosure of contractual agreements that limit such access for investigators	_______21_______
Ancillary and post-trial care	30	Provisions, if any, for ancillary and post-trial care, and for compensation to those who suffer harm from trial participation	________N/A_____
Dissemination policy	31a	Plans for investigators and sponsor to communicate trial results to participants, healthcare professionals, the public, and other relevant groups (eg, via publication, reporting in results databases, or other data sharing arrangements), including any publication restrictions	_________20_____
	31b	Authorship eligibility guidelines and any intended use of professional writers	________20______
	31c	Plans, if any, for granting public access to the full protocol, participant-level dataset, and statistical code	_______N/A______
**Appendices**			
Informed consent materials	32	Model consent form and other related documentation given to participants and authorised surrogates	___Not provided
Biological specimens	33	Plans for collection, laboratory evaluation, and storage of biological specimens for genetic or molecular analysis in the current trial and for future use in ancillary studies, if applicable	______N/A______

*It is strongly recommended that this checklist be read in conjunction with the SPIRIT 2013 Explanation & Elaboration for important clarification on the items. Amendments to the protocol should be tracked and dated. The SPIRIT checklist is copyrighted by the SPIRIT Group under the Creative Commons "Attribution-NonCommercial-NoDerivs 3.0 Unported" license.

For peer review only - http://bmjopen.bmj.com/site/about/guidelines.xhtml

BMJ Open

Implementing a choice of pulmonary rehabilitation models in chronic obstructive pulmonary disease (HomeBase2 trial): protocol for a cluster randomised controlled trial

Journal:	*BMJ Open*
Manuscript ID	bmjopen-2021-057311.R1
Article Type:	Protocol
Date Submitted by the Author:	09-Feb-2022
Complete List of Authors:	Holland, Anne E.; Monash University, Respiratory Research@Alfred, Central Clinical School; Alfred Health, Department of Physiotherapy<br>Jones, Arwel ; Monash University, Respiratory Research@Alfred, Central Clinical School<br>Mahal, Ajay; University of Melbourne, The Nossal Global Institute for Global Health<br>Lannin, Natasha; Monash University, Department of Clinical Neuroscience, Central Clinical School ; Alfred Health, Allied Health (Occupational Therapy)<br>Cox, Narelle; Monash University, Respiratory Research@Alfred, Central Clinical School; Institute for Breathing and Sleep<br>Hepworth, Graham; The University of Melbourne, Statistical Consulting Centre<br>O'Halloran, Paul; La Trobe University, Department of Public Health<br>McDonald, Christine; Austin Health, Department of Respiratory and Sleep Medicine; University of Melbourne, Department of Medicine
**Primary Subject Heading**:	Respiratory medicine
Secondary Subject Heading:	Rehabilitation medicine
Keywords:	Chronic airways disease < THORACIC MEDICINE, REHABILITATION MEDICINE, Emphysema < THORACIC MEDICINE

SCHOLARONE™
Manuscripts

Implementing a choice of pulmonary rehabilitation models in chronic obstructive pulmonary disease (HomeBase2 trial): protocol for a cluster randomised controlled trial

Anne E Holland[1,2,3], Arwel W Jones[1], Ajay Mahal[4], Natasha A. Lannin[5,6] Narelle S Cox[1,3], Graham Hepworth[7], Paul O'Halloran[8], Christine F McDonald[9,10]

1. Respiratory Research@Alfred, Monash University, Melbourne, Australia
2. Department of Physiotherapy, Alfred Health, Melbourne, Australia
3. Institute for Breathing and Sleep, Melbourne, Australia
4. The Nossal Global Institute for Global Health, University of Melbourne, Melbourne, Australia
5. Department of Clinical Neuroscience, Central Clinical School, Monash University, Melbourne, Australia
6. Allied Health (Occupational Therapy), Alfred Health, Melbourne, Victoria, Australia
7. Statistical Consulting Centre, University of Melbourne, Melbourne, Australia
8. Department of Public Health, La Trobe University, Melbourne, Australia
9. Department of Respiratory and Sleep Medicine, Austin Health, Melbourne
10. Department of Medicine, University of Melbourne, Melbourne, Australia

Correspondence to: Prof Anne E Holland; a.holland@alfred.org.au

For peer review only

Authors' contributions

AEH, CFM, AM, NAL and NSC conceived the study; AEH, AWJ, AM, NAL, NSC, GH, PO and CFM developed the protocol and methods; AEH, AWJ, AM, NAL, NSC, GH, PO and CFM prepared the manuscript and approved the final submitted version.

Funding statement

This work is supported by the Commonwealth of Australia Medical Research Future Fund grant MRF1176491. This funding source had no role in the design of this study and will not have any role during its execution, analyses, interpretation of the data, or decision to submit results.

Competing interests

AEH, AM, CFM, GH, NAL, NSC and PO declare grant funding from the Commonwealth of Australia Medical Research Future Fund paid to their institution for the conduct of the trial. CFM declares non-financial board roles as Chair COPD National Program, Lung Foundation Australia and Medical Director Institute for Breathing and Sleep. No other competing interests are declared.

Abstract

Introduction: There is compelling evidence that either centre-based or home-based pulmonary rehabilitation improves clinical outcomes in COPD. There are known health service and personal barriers which prevent potentially eligible patients from accessing the benefits of pulmonary rehabilitation. The aim of this hybrid effectiveness-implementation trial is to examine the effects of offering patients a choice of pulmonary rehabilitation locations (home or centre) compared to offering only the traditional centre-based model

Method and analysis: This is a two-arm cluster randomised, controlled, assessor-blinded trial of 14 centre-based pulmonary rehabilitation services allocated to intervention (offering choice of home-based or centre-based pulmonary rehabilitation) or control (continuing to offer centre-based pulmonary rehabilitation only), stratified by centre-based program setting (hospital vs non-hospital). 490 participants with COPD will be recruited. Centre-based pulmonary rehabilitation will be delivered according to best practice guidelines including supervised exercise training for 8 weeks. At intervention sites, the home-based pulmonary rehabilitation will be delivered according to an established 8-week model, comprising of one home visit, unsupervised exercise training, and telephone calls that build motivation for exercise participation and facilitate self-management. The primary outcome is all-cause, unplanned hospitalisations in the 12 months following rehabilitation. Secondary outcomes include program completion rates and measurements of six-minute walk distance, chronic respiratory questionnaire, EQ-5D-5L, Dyspnoea-12, physical activity and sedentary time at the end of rehabilitation and 12 months following rehabilitation.

For peer review only - http://bmjopen.bmj.com/site/about/guidelines.xhtml

Direct healthcare costs, indirect costs and changes in EQ-5D-5L will be utilised to evaluate cost-effectiveness. A process evaluation will be undertaken to understand how the choice model is implemented and explore sustainability beyond the clinical trial.

Ethics and dissemination: Alfred Hospital Ethics Committee has approved this protocol. The trial findings will be published in peer-reviewed journals, submitted for presentation at conferences and disseminated to patients across Australia with support from national lung charities and societies.

Trial registration number: NCT04217330

Strengths and limitations of this study

- This hybrid effectiveness-implementation trial, conducted across 14 sites, will be the first to compare offering a choice of home-based or centre-based pulmonary rehabilitation to centre-based pulmonary rehabilitation only.
- The trial will only include participants with a diagnosis of COPD.
- All participants, including those in the control group, will have the opportunity to achieve clinical benefits from an evidence-based pulmonary rehabilitation program.
- It is possible that some individuals who are unable or unwilling to attend a centre-based program may choose not to participate in the trial, as the choice of program location is only offered after consent is given. Trial participants may therefore not be representative of all those who might benefit from pulmonary rehabilitation.
- The primary outcome is unplanned hospitalisation, the most valued outcome of patients with COPD.

For peer review only

For peer review only - http://bmjopen.bmj.com/site/about/guidelines.xhtml

Introduction

Chronic obstructive pulmonary disease (COPD) is characterised by persistent respiratory symptoms and airflow limitation.[1] Activity-related dyspnoea and fatigue in COPD ultimately lead to reductions in physical activity and conditioning, which are associated with poor quality of life and exacerbations of respiratory symptoms.[2,3] Acute exacerbations are a leading cause of preventable hospitalisations and account for more than 50% of the costs associated with the treatment of COPD.[4,5,6] The goals of COPD management are to reduce exacerbations and hospitalisations by improving symptoms, optimising self-care and minimising disease progression.[1]

Pulmonary rehabilitation is defined as "a comprehensive intervention based on a thorough patient assessment followed by patient-tailored therapies that include, but are not limited to, exercise training, education, and behaviour change, designed to improve the physical and psychological condition of people with chronic respiratory disease and to promote the long-term adherence to health-enhancing behaviours".[7] Programs have traditionally consisted of twice-weekly attendance as an outpatient in a group setting for 8-12 weeks.[8-9] Clinical trials show that outpatient pulmonary rehabilitation for COPD is highly effective, with Level 1 evidence for improved exercise capacity, reduced breathlessness and improved quality of life, regardless of disease severity.[10] Pulmonary rehabilitation also reduces acute exacerbations, decreases hospital days and reduces hospital admissions, with a concomitant reduction in health care costs.[11-13]

For peer review only - http://bmjopen.bmj.com/site/about/guidelines.xhtml

Despite compelling evidence for the benefits of pulmonary rehabilitation and unequivocal recommendations in COPD guidelines,[8] patient access to pulmonary rehabilitation remains limited worlwide.[14-19] Barriers preventing patients from accessing the benefits of pulmonary rehabilitation occur at a system level (shortage of programs) and individual level (poor physical mobility, distressing symptoms and inability to travel).[20-21] Between 8% and 50% of patients referred to a pulmonary rehabilitation program never attend, and of those who do start, between 10% and 32% do not complete a program.[20] A 2015 American Thoracic Society(ATS)/European Respiratory Society (ERS) policy statement called on the research community to increase access to pulmonary rehabilitation by establishing the efficacy of alternative models.[21]

A low cost model of delivering pulmonary rehabilitation directly into the home was developed (HomeBase) in Australia to improve access and uptake.[22] A previous Phase II randomised controlled equivalence trial[23] showed that HomeBase delivered equivalent clinical outcomes (exercise capacity, symptoms and quality of life) to traditional centre-based rehabilitation in COPD with similar costs and better completion rates (91% vs 49%). The clinical implementation of home-based pulmonary rehabilitation programs has the potential to improve health outcomes, reduce health care utilisation and minimise societal costs but should not be considered a replacement for traditional centre-based programs. Semi-structured interviews with participants who received home-based rehabilitation in the Phase II trial revealed that participants valued the convenience and flexibility of exercise training at home, and the reduced travel burden of a home-based program.[24]

For peer review only - http://bmjopen.bmj.com/site/about/guidelines.xhtml

However, 18% of individuals assessed for eligibility to that trial declined to participate because they wanted to be certain they would receive their preferred choice of a traditional centre-based program. [23]

A recent ATS workshop report identified that the emergence of alternative models of pulmonary rehabilitation pose many unanswered questions for clinical practice, which will be best addressed by prospective clinical implementation trials.[25] At a health service or program level it is likely that the best outcomes and optimal completion rates will be achieved by offering patients a choice of pulmonary rehabilitation locations. Our previous equivalence trial of home versus centre-based rehabilitation showed that patients who completed pulmonary rehabilitation, regardless of group allocation, were 56% less likely to be hospitalised in the following 12 months and had a longer time to their next hospital admission.[23] Offering the choice of home or centre-based pulmonary rehabilitation may increase program completion and therefore result in fewer hospitalisations and more patients with COPD achieving improvements in exercise capacity, breathlessness and quality of life. Until now, the offer of choice of pulmonary rehabilitation location versus traditional centre-based only has not been investigated in a clinical trial.

The aim of this hybrid effectiveness-implementation trial is to examine the effects of offering patients a choice of two pulmonary rehabilitation locations (home or centre) compared to offering only the traditional centre-based model. In order to achieve this aim, the objectives of the trial are threefold:

For peer review only - http://bmjopen.bmj.com/site/about/guidelines.xhtml

162 1) to compare the clinical outcomes, acceptance and completion of pulmonary
163 rehabilitation under the two service models (patient choice between centre-
164 based and home-based, and traditional centre-based only);
165 2) to estimate the costs and compare the cost-effectiveness of the offer of
166 choice of pulmonary rehabilitation location; and
167 3) to understand how the choice model is implemented and explore
168 sustainability beyond the clinical trial.

169 Methods and analysis

170 Design

171 A two-arm cluster randomised, controlled, assessor-blinded trial will be
172 conducted with 14 centre-based pulmonary rehabilitation services across
173 Australia. The overall trial design is depicted in Figure 1. This trial protocol is
174 reported in accordance with SPIRIT guidelines.[26] The trial has been
175 prospectively registered at ClinicalTrials.gov, will be conducted in accordance
176 with the principles of Good Clinical Practice and will be reported according to
177 CONSORT[27] and StaRI[28] standards for cluster randomised controlled trials
178 and implementation studies.

179 Clusters (Pulmonary rehabilitation programs)

180 Included pulmonary rehabilitation services must provide outpatient programs
181 that admit at least 50 people with COPD each year and must be able to
182 demonstrate established protocols for centre-based pulmonary rehabilitation
183 according to national guidelines.[29] These sites are representative of the
184 facilities where pulmonary rehabilitation is delivered in Australia, including
185 community programs, and large and medium sized hospitals.

For peer review only - http://bmjopen.bmj.com/site/about/guidelines.xhtml

186 **Randomisation**

187 Eligible pulmonary rehabilitation services will be randomised 1:1 to either the intervention group (offering choice of home-based or centre-based pulmonary rehabilitation) or control group (continuing to offer only centre-based pulmonary rehabilitation) stratified by setting of existing centre-based pulmonary rehabilitation (hospital vs non-hospital). Such strata have been chosen to balance for potential factors related to implementation of choice of rehabilitation models (e.g. local staff capacity and infrastructure) or differences in sociodemographic profiles in areas where hospital or non-hospital programs are usually situated (e.g. urban vs rural)[30] and most common reasons affecting attendance at centre-based rehabilitation or uptake of home-based rehabilitation (e.g. accessibility of/ distance to hospital venues).[24,31] Randomisation (sequence generation and allocation concealment) will be performed using a secure online randomisation service managed by an independent agency. Block randomisation is being used to both balance cluster allocation within strata and to help ensure the methods (in particular the block size) can remain consistent throughout the trial (e.g. should clusters withdraw from the trial).

For peer review only - http://bmjopen.bmj.com/site/about/guidelines.xhtml

Blinding

Research staff: Site principal investigators at each cluster will be notified of their allocation by the research team. Clinicians within sites who are involved in delivery of the intervention will not be blinded to group allocation. However, research staff within clusters that conduct post-intervention assessments and enter questionnaire data into the trial database will be blinded to group allocation.

Participants: Participants will be blinded to group allocation. Patients will be informed that this study is examining the impact of pulmonary rehabilitation models on patient and health system outcomes. The specific nature of the models of interest, or the intervention group to which their site has been allocated will not be disclosed. As this is a study of behaviour (the impact of offering choice of pulmonary rehabilitation models), limited disclosure of the study aim regarding comparison of models is necessary. To truly understand and measure the impact of offering choice of program models, the offer of choice in the intervention groups will be made after participant consent for the trial has been obtained, so that the choice can be recorded.

Participants

To be eligible for inclusion at the 14 pulmonary rehabilitation services participants must i) have a diagnosis of COPD confirmed on spirometry; ii) be able to read, write and speak English; iii) be able to provide informed consent. Exclusion criteria include: i) attendance at pulmonary rehabilitation in the last 12 months; ii) comorbidities which preclude exercise training (with reference to absolute and relative contraindications for field walking tests)[32]; iii) inability to provide informed consent.

For peer review only - http://bmjopen.bmj.com/site/about/guidelines.xhtml

Recruitment

Eligible participants will be identified through referrals to pulmonary rehabilitation programs at participating clusters. To ensure that referrals are maximised across all sites, respiratory and general physicians at their affiliated hospitals will receive three-monthly reminders to refer patients with COPD to pulmonary rehabilitation, consistent with current guidelines for best practice care (COPD-X).[33] Screening will be conducted by the site investigator, who is a staff member in the pulmonary rehabilitation program. Potential participants will be sent an information flier about the study prior to their initial pulmonary rehabilitation appointment. They will be invited to discuss the study further at their initial appointment, or by telephone.

Delivery of pulmonary rehabilitation

Participants in both groups will receive a copy of the 'Better Living with Exercise' booklet, produced by Lung Foundation Australia. The 'Better Living with Exercise' booklet assists people with COPD to work with their health professionals to optimise their exercise program, based on their individual health conditions, goals and interests. All participants will also receive details to access an online version of the 'Better Living with COPD; A Patient Guide' on the Lung Foundation Australia website. The 'Better Living with COPD: A Patient Guide' (Third Edition)[34] was developed to support COPD patients to better understand and manage their condition. At the completion of the rehabilitation period participants will follow usual care at the trial site which may or may not include a referral to a locally available supervised exercise maintenance program to promote ongoing exercise adherence. The number of participants who opt to join maintenance programmes will be recorded.

For peer review only - http://bmjopen.bmj.com/site/about/guidelines.xhtml

Control group*:* Pulmonary rehabilitation programs assigned to the control group will offer eligible participants the opportunity to participate in their usual centre-based pulmonary rehabilitation, delivered according to current best practice guidelines.[29] This will be an 8-week, twice weekly outpatient group-based supervised program, with individually prescribed exercise training and self-management education. At least 30 minutes of aerobic exercise training will be performed each session, plus resistance exercises using functional activities such as stair climbing and sit-to-stand practice, as well as free weights for the upper limbs. Participants will also be encouraged to exercise at home on 3 occasions each week and to record this in the Lung Foundation Australia 'Better Living with Exercise' resource diary. Programs may also include self-management training in structured (lecture-based) and unstructured disease management education on a group and/or 1:1 basis, in accordance with existing clinical practice at the trial site.

Intervention group: Pulmonary rehabilitation programs assigned to the intervention group will offer eligible participants the choice of participating in an 8 week program of either home-based or traditional centre-based pulmonary rehabilitation (see Control group). Home-based rehabilitation will be delivered according to our protocol (HomeBase), which has been published in detail previously.[22,23,35] Briefly, HomeBase includes the following components:

Home Visit: The program commences with one home visit by a physiotherapist experienced in pulmonary rehabilitation during which exercise goals are established, the first exercise session is supervised and aspects of

For peer review only

278 the self-management program that cannot be adequately dealt with during subsequent telephone calls (e.g. review of inhaler technique), are covered.

280 *Exercise training*: Participants will follow an aerobic and strength training program, which will be supervised for the first session and unsupervised thereafter. For the aerobic training, a walking program will be prescribed with participants set a walking distance or number of steps to be completed in a given time via a pedometer and on the basis of a baseline 6MWT (initial target intensity of 80% of the speed walked in the test). At least 30 minutes of aerobic exercise is recommended for each session, for at least 5 sessions per week. Strength training will utilise functional activities and equipment that are readily accessible in the home environment (e.g. sit-to-stand from a dining chair, water bottles for upper limb weights). Participants in the intervention group who choose home-based pulmonary rehabilitation will not have to return their pedometers at end of the program and can use the device for the entire study duration.

293 *Home Exercise diary*: The initial goals set for aerobic and resistance exercise and details of the supervised exercise session during the home visit will be recorded in a home exercise diary. Participants will be encouraged to document all subsequent program goals (exercise or health) and completed unsupervised exercise sessions, including duration and distance walked and the number and type of resistance exercises performed, in their diary each week. We have shown a significant relationship between diary-documented exercise sessions and device measured physical activity.[36] Participants will also be asked to record what barriers may prevent them from achieving their goals or exercise and how they anticipate they may overcome these barriers.

For peer review only - http://bmjopen.bmj.com/site/about/guidelines.xhtml

303 Prior to commencing each unsupervised exercise session, the home exercise diary prompts the participant to check whether they have any symptoms of moderate or severe exacerbation and to contact the pulmonary rehabilitation clinician at the site if symptomatic.

Weekly telephone calls: Following the home visit in week one, each participant will receive seven once-weekly telephone calls. All calls will be delivered using a motivational interviewing approach[37] by a physiotherapist experienced in pulmonary rehabilitation and who has undertaken undertake formal training in motivational interviewing for health care. Additional trial-based training and regular fidelity checks using the Motivational Interviewing Treatment Integrity Scale version 4.2.1[38] will also take place. During the telephone calls the physiotherapist will review the home exercise diary; progress the exercise prescription; review symptoms and facilitate self-management of exacerbations; and deliver self-management education via scripted telephonic modules. These structured telephone modules will be used to explore and build motivation for exercise participation.[22,23] The home exercise diary provides participants with a menu of topics related to self-care. Participants will be encouraged to select a topic they feel is relevant to them for discussion with the physiotherapist in their weekly call, providing opportunities for self-management education and goal setting. All participants will have a discussion on managing an acute exacerbation of their COPD and long-term exercise planning, consistent with the goals of centre-based pulmonary rehabilitation programs. Participants will have access to the 'Better Living with COPD: A Patient Guide' and the C.O.P.E program (COPD Online Patient Education) to support topic discussions.

For peer review only

Data collection and follow up

Each participant will complete 8 weeks of rehabilitation and a follow-up of 12 months. Outcomes will be assessed at baseline, end of rehabilitation (after 8 weeks) and 12 months following the end of rehabilitation. We will undertake assessments with all participants (unless not possible e.g. due to death, loss-to follow up, study withdrawal) at 8 weeks and 12 months irrespective of whether a participant has completed their pulmonary rehabilitation program. Participants will record any health care utilisation during the 12 months follow-up (visits to health professionals, hospitalisations) in a diary. Each participant will be telephoned monthly during the 12 months by site research staff to encourage diary completion and capture patient-reported information on health-care utilisation. These calls represent an additional contact with patients that would not be provided as part of routine clinical practice at trial sites, but the purpose of the call is only for research staff to support collection of data on health-care use. Hospitalisation and use of other hospital services will be confirmed by medical record audit at 12 months, whilst 12-month medical and pharmaceutical resource use will be obtained from Services Australia using Pharmaceutical Benefits Scheme (PBS) and Medicare Benefits Schedule (MBS) data. Consent will be sought to use participants' PBS and MBS data at the time of consent to the study. The schedule for assessments and follow-up is provided in Table 1.

Outcomes

Primary Outcome

All-cause, non-elective hospitalisation over 12 months: The number of participants admitted to hospital as an inpatient at least once in the 12 months

For peer review only - http://bmjopen.bmj.com/site/about/guidelines.xhtml

following pulmonary rehabilitation will be compared between groups. Avoidance of hospitalisation is the research outcome that people with COPD value most highly and is of critical importance to the health system.[39] We have chosen all cause, non-elective hospitalisation due to the challenges in assessing whether an admission is COPD-related in a group where multimorbidity is ubiquitous (median 4 coexisting conditions in our previous trial[23]) and the potential for bias in adjudication of respiratory-related admissions significant. We have developed an effective method to collect these data in our previous trial[23] combining medical record audit with monthly telephone calls to participants, to ensure comprehensive coverage of health care utilisation.

Secondary outcomes

Program completion: The number of participants who complete their allocated pulmonary rehabilitation program will be compared between groups at 8 weeks. Completion is defined as undertaking 70% of planned sessions in accordance with recent recommendations.[25]

6-minute walk distance (6MWD): The 6MWD is a validated measure of functional exercise capacity for COPD.[32] The 6MWD predicts both future hospitalisation[40] and survival.[41] It is responsive to change following pulmonary rehabilitation[10] and is an outcome that matters to patients.[42] The test will be performed in accordance with international standards,[32] including two tests at each time point, with the better (i.e. longest distance) 6MWD recorded.

Chronic Respiratory Questionnaire (CRQ): The CRQ is a disease-specific health-related quality of life measure with domains of dyspnoea, fatigue, mastery and emotional function. It is a patient-centred outcome that is

For peer review only

For peer review only - http://bmjopen.bmj.com/site/about/guidelines.xhtml

responsive to change following pulmonary rehabilitation.[10] The self-reported version of the CRQ[43] will be used.

EQ-5D-5L: This validated generic quality of life measure[44] is used to estimate health benefits in terms of quality-adjusted life-years (QALYs),[45] and is recommended for economic analyses.

Dyspnoea-12: This a global measure of breathlessness severity that captures both the physical and affective components of dyspnoea. It has excellent internal consistency, validity and reliability in COPD.[46]

Physical activity participation: This is an important measure of behaviour change following pulmonary rehabilitation. We will measure time spent in moderate to vigorous physical activity and sedentary time, using the ActiGraph GT3X, a waist-worn, tri-axial accelerometer that has been validated in people with COPD.[47] Seven days of monitoring (in the week following the 8 week or 12 month assessment) will be performed to allow for at least 4 valid days of at least 8 hours wear time.[48]

Health care utilisation across 12 months: Healthcare utilisation will be recorded monthly in participant diaries and telephone record sheets kept by site research staff. We will confirm health care utilisation from hospital records, MBS and PBS data.

Sample size

The primary outcome is unplanned hospitalisation (inpatient admissions) in the 12 months following pulmonary rehabilitation. In our published Phase II trial,[23] 57% of the centre-based group were free from hospitalisation at 12 months, whereas 78% of rehabilitation completers were free from hospitalisation (regardless of group). Completion was 91% for HomeBase,

For peer review only

For peer review only - http://bmjopen.bmj.com/site/about/guidelines.xhtml

and 49% for centre-based.[23] The novel aspect of the current study is that intervention participants will be offered a choice of models. Based on our experience recruiting for HomeBase, we expect that 75% of individuals may select a home program of whom 91% will complete, and 25% will select centre-based of whom 49% will complete. We thus anticipate a completion rate of 80% in the intervention (choice) group.

At 80% completion, our data indicate that 72% of the intervention group would remain free from admission at 12 months, compared to the observed rate of 57% in centre-based (control) participants.[23] We thus expect 15% reduction in hospitalisations in the intervention group (choice of home-based or centre-based rehabilitation) compared to control (centre-based rehabilitation only). To detect a difference in remaining free from hospitalisation between intervention and control groups of 15% (72% vs 57%) with 80% power and a 2-sided 0.05 significance level, 312 participants (156 in each group) are required. Adjusting for clustering by program (intra-cluster correlation coefficient of 0.01, based on our published 2-site trial, and 35 participants per cluster), the total required sample is 418. Our previous study had 10% loss to follow-up, so to allow for this we will recruit a total of 490 participants from 14 pulmonary rehabilitation programs.

Statistical analysis

Generalised linear mixed models will be used to analyse the primary outcome of hospitalisation, and linear mixed models will be used for continuous outcomes. These will account for correlation between participants within a cluster. Analyses will be based on intention-to-treat and the level of significance will be set at $p < 0.05$.

For peer review only - http://bmjopen.bmj.com/site/about/guidelines.xhtml

Economic Evaluation

Economic evaluation will follow the protocol developed for our Phase II trial.[22,23]

Cost comparisons: Cost comparisons will include comparison of per person costs, including direct (health system) and indirect (personal) health care costs for the two groups. Direct costs will include staff time, consumables, communications and overheads. Intervention costs will include staff inputs by duration, type and resource use (including troubleshooting and support) and equipment. Personal costs will include transportation, travel time and impact of the intervention on the economic activities of other household members. Health system costs will include visits to the general practitioner, specialist or emergency department; use of chronic disease services; and hospitalisation.

Cost-effectiveness analysis: An incremental cost-effectiveness analysis will be undertaken to compare differences in costs between the two groups with differences in quality adjusted life years (QALYs) and number of hospitalisations in the 12-month follow-up period. For the former, a utility index (UI) will be calculated from the EQ-5D-5L by applying a 'social tariff', which then enables estimation of health benefits in terms of QALYs. The conversion to QALYs will be based on the assumption that for health states measured at successive points of time, the duration of each health state is exactly one half of the time interval between the two measurements.[49] For the latter, the indicator of cost effectiveness can be interpreted as the incremental cost of avoiding a hospitalisation.

For peer review only - http://bmjopen.bmj.com/site/about/guidelines.xhtml

451 **Process evaluation**

452 A process evaluation, informed by the Medical Research Council Guidance on
453 Process Evaluations of complex interventions,[50] is also planned for this trial.
454 Offering a choice of program models represents a departure from traditional
455 care. Thus, understanding both clinician and patient experiences of this new
456 approach is critical to guide scale-up and future implementation. A detailed
457 protocol for the process evaluation will be published separately. Briefly, the
458 process evaluation will undertake a theory-based approach using the
459 Theoretical Domains Framework (TDF).[51] The deductive coding using the
460 TDF will allow the team to understand clinician or patient behaviours that act
461 as barriers or facilitators to implementation of HomeBase, and to develop a
462 compendium of implementation strategies for future scale-up. Implementation
463 will be further explored using the RE-AIM (Reach, Effectiveness, Adoption,
464 Implementation, and Maintenance) framework[52] with collection of both
465 quantitative and qualitative data throughout the trial (Table 2) to provide
466 insights into translation and behaviour change success in intervention
467 clusters.

468 **Ethics and dissemination**

469 This protocol (version 2, 16 August 2020) has received approval from Alfred
470 Hospital Human Research Ethics Committee (379/19). Local research ethics
471 committee approvals have also been obtained at sites where the National
472 Mutual Acceptance scheme does not apply. Each site is also required to
473 obtain local governance approval prior to commencement of data collection.
474 Site investigators will be informed by the trial coordinator of any necessary
475 modifications to the protocol and amendments to local ethics and/or

For peer review only

governance approval. Potential participants at each site may be in a dependent relationship with pulmonary rehabilitation staff. For this reason pulmonary rehabilitation staff will only provide initial information about the trial to a potential participant, either at the time of initial assessment or over the telephone. If the potential participant is interested in receiving further detailed information, or providing informed consent, a researcher who is not in a dependent relationship with the potential participant will undertake this. The trial findings will be published in peer-reviewed journals and submitted to national and international conferences for presentation. The authorship of any publications will adhere to the guidelines established by the International Committee of Medical Journal Editors. We will disseminate results to patients with COPD across Australia through Lung Foundation Australia's e-newsletter and website, and to health professionals through Thoracic Society of Australia and New Zealand's e-news, scientific conferences and webinar program.

Safety monitoring and reporting

All sites will maintain an adverse event log for the trial period. An adverse event in the trial is being defined as any unfavourable, unintended diagnosis, symptom, sign (including an abnormal laboratory finding), syndrome, or disease that occurs during the trial, having been absent at baseline, or if present at baseline, appears to have worsened. A serious adverse event is defined as any adverse event leading to i) death; ii) serious deterioration in health resulting in a life-threatening illness or injury, or permanent impairment of a body structure or a body function; iii) hospitalisation or prolongation of existing hospitalisation; iv) medical or surgical intervention to prevent life-threatening illness; v) foetal distress, foetal death or a congenital abnormality.

For peer review only

For peer review only - http://bmjopen.bmj.com/site/about/guidelines.xhtml

Sites will inform the trial coordinator of any adverse event that necessitates a change to the protocol or participant information and consent form, is unexpected, or any serious adverse events. The trial coordinator will ensure the data and safety monitoring board (DSMB), relevant research ethics committees or governance offices are informed of any serious adverse events where necessary. The DSMB, consisting of a respiratory physician, two physiotherapists and a statistician, will make the final decision on the relatedness of any adverse events to the trial interventions or procedures. Unless otherwise determined by trial events, the DSMB will convene bi-annually.

Data management

Hard copy original data collection forms will be stored in a locked filing cabinet within a locked office at each trial site. Electronic data will be stored centrally in a purpose-built on-line database (www.adeptrs.com), with encryption and password protection. No identifying information will be stored in the online database. Electronic data for all sites will be accessible by the coordinating principal investigator and the trial coordinator. Site specific investigators will only have access to data relating to their individual site.

For peer review only

Patient and public involvement

The experiences of HomeBase participants in our previous Phase II trial[24] were critical to formulating the current proposal. Hospitalisation has been chosen as the primary outcome of this trial because it is the research outcome that is most meaningful for patients with COPD.[39] Two patients with COPD will sit on the trial steering committee. All patient-facing trial resources (eg fliers, Participant Information and Consent Forms, diaries) have undergone review

For peer review only - http://bmjopen.bmj.com/site/about/guidelines.xhtml

by patients with COPD via Lung Foundation Australia's COPD Patient Advisory Group.

Trial status

Trial recruitment began in March 2021 and is ongoing.

Discussion

This research will be the first randomised controlled trial to determine the clinical benefits and health care costs of offering patients a choice of two pulmonary rehabilitation locations (home- or centre-based) compared to offering only traditional centre-based models. Until recently, there has been very little "choice" for patients referred to pulmonary rehabilitation; the delivery model of centre-based pulmonary rehabilitation has largely been a "one size fits all" that is inconsistent with the personalised approach of modern medicine.[25]

For peer review only

The adoption of alternative models for pulmonary rehabilitation will rely on demonstration of comparable or better clinical outcomes compared with those of traditional pulmonary rehabilitation programs, which can be delivered in a cost-effective manner.[53] A novel aspect of this trial is that our intervention will offer patients with COPD the choice of two rehabilitation models that are underpinned by strong efficacy data, an approach that provides patients with increased opportunities to attain the benefits of pulmonary rehabilitation. Our previous economic analysis[54] suggested that completion of pulmonary rehabilitation is an independent predictor of lower health care costs in COPD.

Based on head-to-head trial evidence of home versus outpatient-based pulmonary rehabilitation, the 2017 Australian Pulmonary Rehabilitation

For peer review only - http://bmjopen.bmj.com/site/about/guidelines.xhtml

guidelines recommended home programs as an alternative to hospital-based programs.[29] The offer of a home-based program has long been a limited option in pulmonary rehabilitation services.[16] The coronavirus disease (COVID-19) pandemic had led to many pulmonary rehabilitation services rapidly transitioning to home-based models but there remains little information about how best to implement these models in a clinical setting.[25] Home-based programs have the potential to address many of the patient-related and system-related barriers to attending traditional centre-based pulmonary rehabilitation models in COPD including improvements to access (e.g. greater scope in program delivery) and uptake (allowing patient preference for home-based care, reducing barriers related to travel and disability). Targeted implementation is required to translate the clinical efficacy of home-based programs to important health outcomes in routine practice. Implementation of home-based programs alongside the traditional outpatient centre-based models may allow evidence-based pulmonary rehabilitation to be more accessible to patients. A process evaluation will be conducted alongside the cluster randomised controlled trial in order to understand barriers and facilitators to offering choice of pulmonary rehabilitation models at trial sites, and to provide data that will guide future implementation.

This trial will provide the evidence needed to underpin future guidelines and policy decisions for pulmonary rehabilitation. If successful, the findings will inform a key change in the future of pulmonary rehabilitation services worldwide, where delivery includes more choices for patients and opportunities for greater personalisation of programs.

For peer review only - http://bmjopen.bmj.com/site/about/guidelines.xhtml

References

1. Global Strategy for the Diagnosis, Management, and Prevention of COPD 2021 report, Global Initiative for Chronic Obstructive Lung Disease (GOLD) [Accessed 4 June 2021]. Available from: https://goldcopd.org/2021-gold-reports/
2. Hanania NA, O'Donnell DE. Activity-related dyspnea in chronic obstructive pulmonary disease: physical and psychological consequences, unmet needs, and future directions. *Int J Chron Obstruct Pulmon Dis* 2019;14:1127-1138.
3. Spruit MA, Burtin C, De Boever P, Langer D, Vogiatzis I, Wouters EF, Franssen FM. COPD and exercise: does it make a difference? *Breathe* 2016;12(2):38-49.
4. Australian Institute of Health and Welfare 2017. Web Update: Potentially preventable hospitalisations in 2015-16 [Accessed 28 December 2018]. Available from: https://www.myhealthycommunities.gov.au/our-reports/potentially-preventable-hospitalisations-update/july-2017 =
5. Miravitlles M, Garcia-Polo C, Domenech A, et al. Clinical outcomes and cost-analysis of exacerbations in chronic obstructive pulmonary disease. *Lung* 2013; 191:523-530
6. Wouters EF. Economic analysis of the Confronting COPD survey: an overview of results. *Respir Med* 2003; 97:S3-14
7. Spruit M, Singh S, Garvey C, *et al*. An official American Thoracic Society/European Respiratory Society statement: key concepts and advances in pulmonary rehabilitation. *Am J Resp Crit Care Med* 2013;188:13-64.
8. Holland AE, Cox NS, Houchen-Wolloff L, Rochester CL, Garvey C, ZuWallack R, Nici L, Limberg T, Lareau SC, Yawn BP, Galwicki M, Troosters T, Steiner M, Casaburi R, Clini E, Goldstein RS, Singh SJ. Defining Modern Pulmonary Rehabilitation. An Official American Thoracic Society Workshop Report. *Ann Am Thorac Soc* 2021;18(5):e12-e29.
9. Spruit MA, Pitta F, Garvey C, ZuWallack RL, Roberts CM, Collins EG, et al.; ERS Rehabilitation and Chronic Care, and Physiotherapists Scientific Groups; American Association of Cardiovascular and Pulmonary Rehabilitation; ATS Pulmonary Rehabilitation Assembly and the ERS COPD Audit team. Differences in content and organisational aspects of pulmonary rehabilitation programmes. *Eur Respir J* 2014;43:1326–1337.
10. McCarthy B, Casey D, Devane D, *et al*. Pulmonary rehabilitation for chronic obstructive pulmonary disease. *Cochrane Database Syst Rev* 2015;23
11. Griffiths TL, Phillips CJ, Davies S, Burr ML, Campbell IA. Cost effectiveness of an outpatient multidisciplinary pulmonary rehabilitation programme. *Thorax* 2001; 56: 779-84

For peer review only - http://bmjopen.bmj.com/site/about/guidelines.xhtml

12. Guell R, Casan P, Belda J, Sangenis M, Morante F, Guyatt GH, Sanchis J. Long-term effects of outpatient rehabilitation of COPD: A randomized trial. *Chest* 2000; 117: 976-83.

13. Moore E, Palmer T, Newson R, Majeed A, Quint JK, Soljak MA. Pulmonary Rehabilitation as a Mechanism to Reduce Hospitalizations for Acute Exacerbations of COPD: A Systematic Review and Meta-Analysis. *Chest* 2016;150(4):837-859.

14. Brooks D, Sottana R, Bell B, Hanna M, Laframboise L, Selvanayagarajah S, Goldstein R. Characterization of pulmonary rehabilitation programs in Canada in 2005. Can Respir J 2007;14(2):87-92

15. Camp PG, Hernandez P, Bourbeau J, Kirkham A, Debigare R, Stickland MK, Goodridge D, Marciniuk DD, Road JD, Bhutani M, Dechman G. Pulmonary rehabilitation in Canada: A report from the Canadian Thoracic Society COPD Clinical Assembly. *Can Respir J* 2015;22(3):147-52.

16. Marks G, Reddel H, Guevara-Rattray E, Poulos L, Ampon R. Monitoring pulmonary rehabilitation and long-term oxygen therapy for people with chronic obstructive pulmonary disease (COPD) in Australia: a discussion paper. Australian Institute of Health and Welfare, Canberra, 2013 [Accessed 4 June 2021] Available from: https://www.aihw.gov.au/reports/chronic-respiratory-conditions/monitoring-pulmonary-rehabilitation-and-long-term/contents/table-of-contents.

17. Nishi SP, Zhang W, Kuo YF, Sharma G. Pulmonary Rehabilitation Utilization in Older Adults With Chronic Obstructive Pulmonary Disease, 2003 to 2012. *J Cardiopulm Rehabil Prev* 2016;36(5):375-82.

18. Steiner M, Holzhauer-Barrie J, Lowe D, Searle L, Skipper E, Welham S, Roberts CM. Pulmonary Rehabilitation: Time to breathe better. National Chronic Obstructive Pulmonary Disease (COPD) Audit Programme: Resources and organisation of Pulmonary Rehabilitation services in England and Wales 2015. National organisational audit report. Royal College of Physicians, London, 2015 [Accessed 4 June 2021]. Available from: https://www.rcplondon.ac.uk/projects/outputs/pulmonary-rehabilitation-time-breathe-better

19. Yohannes AM, Connolly MJ. Pulmonary rehabilitation programmes in the UK: a national representative survey. *Clin Rehabil* 2004;18(4):444-9.

20. Keating A, Lee A, Holland AE. What prevents people with chronic obstructive pulmonary disease from attending pulmonary rehabilitation? A systematic review. *Chron Respir Dis* 2011; 8: 89-99.

21. Rochester CL, Vogiatzis I, Holland AE, Lareau SC, Marciniuk DD, Puhan MA, Spruit MA, Masefield S, Casaburi R, Clini EM, Crouch R, Garcia-Aymerich J, Garvey C, Goldstein RS, Hill K, Morgan M, Nici L, Pitta F, Ries AL, Singh SJ, Troosters T, Wijkstra PJ, Yawn BP, ZuWallack RL; ATS/ERS Task Force on Policy in Pulmonary Rehabilitation. An Official American Thoracic Society/European Respiratory Society Policy Statement: Enhancing Implementation, Use, and Delivery of Pulmonary Rehabilitation. *Am J Respir Crit Care Med* 2015;192(11):1373-86.

For peer review only - http://bmjopen.bmj.com/site/about/guidelines.xhtml

22. Holland AE, Mahal A, Hill CJ, Lee AL, Burge AT, Moore R, Nicolson C, O'Halloran P, Cox NS, Lahham A, Ndongo R, Bell E, McDonald CF. Benefits and costs of home-based pulmonary rehabilitation in chronic obstructive pulmonary disease - a multi-centre randomised controlled equivalence trial. *BMC Pulm Med* 2013;13:57.

23. Holland AE, Mahal A, Hill CJ, Lee AL, Burge AT, Cox NS, Moore R, Nicolson C, O'Halloran P, Lahham A, Gillies R, McDonald CF. Home-based rehabilitation for COPD using minimal resources: a randomised, controlled equivalence trial. *Thorax* 2017;72(1):57-65.

24. Lahham A, McDonald CF, Mahal A, Lee AL, Hill CJ, Burge AT, Cox NS, Moore R, Nicolson C, O'Halloran P, Gillies R, Holland AE. Home-based pulmonary rehabilitation for people with COPD: A qualitative study reporting the patient perspective. *Chron Respir Dis* 2018;15(2):123-130.

25. Holland AE, Cox NS, Houchen-Wolloff L, Rochester CL, Garvey C, ZuWallack R, Nici L, Limberg T, Lareau SC, Yawn BP, Galwicki M, Troosters T, Steiner M, Casaburi R, Clini E, Goldstein RS, Singh SJ. Defining Modern Pulmonary Rehabilitation. An Official American Thoracic Society Workshop Report. *Ann Am Thorac Soc* 2021;18(5):e12-e29.

26. Chan AW, Tetzlaff JM, Gøtzsche PC, Altman DG, Mann H, Berlin JA, Dickersin K, Hróbjartsson A, Schulz KF, Parulekar WR, Krleza-Jeric K, Laupacis A, Moher D. SPIRIT 2013 explanation and elaboration: guidance for protocols of clinical trials. *BMJ* 2013;346:e7586.

27. Campbell M K, Piaggio G, Elbourne D R, Altman D G. Consort 2010 statement: extension to cluster randomised trials *BMJ* 2012;345 :e5661.

28. Pinnock H, Barwick M, Carpenter CR, Eldridge S, Grandes G, Griffiths CJ, Rycroft-Malone J, Meissner P, Murray E, Patel A, Sheikh A, Taylor SJ, Sta RIG. Standards for Reporting Implementation Studies (StaRI) Statement. *BMJ* 2017; 356: i6795

29. Alison JA, McKeough ZJ, Johnston K, McNamara RJ, Spencer LM, Jenkins SC, Hill CJ, McDonald VM, Frith P, Cafarella P, Brooke M, Cameron-Tucker HL, Candy S, Cecins N, Chan AS, Dale MT, Dowman LM, Granger C, Halloran S, Jung P, Lee AL, Leung R, Matulick T, Osadnik C, Roberts M, Walsh J, Wootton S, Holland AE. Australian and New Zealand Pulmonary Rehabilitation Guidelines. *Respirology* 2017; 22 (4):800–819.

30. Terry DR, Nguyen H, Kim JA, Islam MR. Factors contributing to COPD hospitalisations from 2010 to 2015: Variation among rural and metropolitan Australians. *Clin Respir J*. 2019;13(5):306-313.

31. Keating A, Lee A, Holland AE. What prevents people with chronic obstructive pulmonary disease from attending pulmonary rehabilitation? A systematic review. *Chron Respir Dis*. 2011;8(2):89-99.

32. Holland AE, Spruit MA, Troosters T, Puhan MA, Pepin V, Saey D, McCormack MC, Carlin BW, Sciurba FC, Pitta F, Wanger J, MacIntyre N, Kaminsky DA, Culver BH, Revill SM, Hernandes NA, Andrianopoulos V, Camillo CA, Mitchell KE, Lee AL, Hill CJ, Singh SJ. An official European Respiratory Society/American Thoracic Society technical standard: field

For peer review only - http://bmjopen.bmj.com/site/about/guidelines.xhtml

walking tests in chronic respiratory disease. Eur Respir J. 2014;44(6):1428-46.

33. Yang IA, George J, McDonald CF, McDonald V, O'Brien M, Smith B, Zwar N, Dabscheck E. The COPD-X Plan: Australian and New Zealand Guidelines for the management of Chronic Obstructive Pulmonary Disease 2021. Version 2.63, February 2021.

34. Queensland Health Statewide Respiratory Clinical Network and Lung Foundation Australia. Better Living with Chronic Obstructive Pulmonary Disease A Patient Guide. 3rd ed, Queensland, 2016.

35. HomeBase Rehab [Accessed 4 June 2021]. Available from: https://homebaserehab.net/

36. Lahham A, McDonald CF, Mahal A, Lee AL, Hill CJ, Burge AT, Cox NS, Moore R, Nicolson C, O'Halloran P, Gillies R, Holland AE. Acceptability and validity of a home exercise diary used in home-based pulmonary rehabilitation: A secondary analysis of a randomised controlled trial. *Clin Respir J* 2018; 12: 2057-64.

37. Miller WR, Rollnick S R. Motivational interviewing: Helping people change. 3rd ed. NY: Guildford Press 2013.

38. Moyers TB, Rowell LN, Manuel JK, et al. The Motivational Interviewing Treatment Integrity Code (MITI 4): Rationale, Preliminary Reliability and Validity. J Subst Abuse Treat 2016;65:36-42. doi: 10.1016/j.jsat.2016.01.001 [published Online First: 2016/02/15]

39. Zhang Y, Morgan RL, Alonso-Coello P, Wiercioch W, Bala MM, Jaeschke RR, Styczen K, Pardo-Hernandez H, Selva A, Ara Begum H, Morgano GP, Waligora M, Agarwal A, Ventresca M, Strzebonska K, Wasylewski MT, Blanco-Silvente L, Kerth JL, Wang M, Zhang Y, Narsingam S, Fei Y, Guyatt G, Schunemann HJ. A systematic review of how patients value COPD outcomes. *Eur Respir J* 2018; 52.

40. Spruit MA, Polkey MI, Celli B, Edwards LD, Watkins ML, Pinto-Plata V, Vestbo J, Calverley PM, Tal-Singer R, Agusti A, Coxson HO, Lomas DA, Macnee W, Rennard S, Silverman EK, Crim CC, Yates J, Wouters EF. Predicting Outcomes from 6-Minute Walk Distance in Chronic Obstructive Pulmonary Disease. *J Am Med Dir Assoc* 2012; 13: 291-7.

41. Cote CG, Pinto-Plata V, Kasprzyk K, Dordelly LJ, Celli BR. The 6-min walk distance, peak oxygen uptake, and mortality in COPD. *Chest* 2007; 132: 1778-85.

42. Holland AE, Hill CJ, Rasekaba T, Lee A, Naughton MT, McDonald CF. Updating the minimal important difference for six-minute walk distance in patients with chronic obstructive pulmonary disease. *Arch Phys Med Rehabil* 2010; 91: 221-5.

43. Williams J, Singh S, Sewell L, Guyatt GH, Morgan MDL. Development of a self-reported Chronic Respiratory Questionnaire (CRQ-SR). *Thorax* 2001; 56: 954-959.

44. Nolan CM, Longworth L, Lord J, Canavan JL, Jones SE, Kon SS, Man WD. The EQ-5D-5L health status questionnaire in COPD: validity,

For peer review only - http://bmjopen.bmj.com/site/about/guidelines.xhtml

responsiveness and minimum important difference. *Thorax* 2016;71(6):493-500.

45. Herdman M, Gudex C, Lloyd A, Janssen M, Kind P, Parkin D, Bonsel G, Badia X. Development and preliminary testing of the new five-level version of EQ-5D (EQ-5D-5L). *Qual Life Res* 2011;20(10):1727-36.

46. Yorke J, Moosavi SH, Shuldham C, Jones PW. Quantification of dyspnoea using descriptors: development and initial testing of the Dyspnoea-12. *Thorax* 2010; 65: 21-6.

47. Van Remoortel H, Raste Y, Louvaris Z, Van Remoortel, H., Raste, Y., Louvaris, Z., Giavedoni, S., Burtin, C., Langer, D., Wilson, F., Rabinovich, R., Vogiatzis, I., Hopkinson, N. S., Troosters, T., & PROactive consortium. Validity of six activity monitors in chronic obstructive pulmonary disease: a comparison with indirect calorimetry. *PLoS One* 2012; **7**: e39198.

48. Demeyer H, Mohan D, Burtin C, Vaes AW, Heasley M, Bowler RP, Casaburi R, Cooper CB, Corriol-Rohou S, Frei A, Hamilton A, Hopkinson NS, Karlsson N, Man WD, Moy ML, Pitta F, Polkey MI, Puhan M, Rennard SI, Rochester CL, Rossiter HB, Sciurba F, Singh S, Tal-Singer R, Vogiatzis I, Watz H, Lummel RV, Wyatt J, Merrill DD, Spruit MA, Garcia-Aymerich J, Troosters T; Chronic Lung Disease Biomarker and Clinical Outcome Assessment Qualification Consortium Task Force on Physical Activity. Objectively Measured Physical Activity in Patients with COPD: Recommendations from an International Task Force on Physical Activity. Chronic Obstr Pulm Dis. 2021;8(4):528-550.

49. Sinnott PL, Joyce VR, Barnett PG. Preference Measurement in Economic Analysis. Guidebook. Health Economics Resource Center, Menlo Park CA. VA Palo Alto, 2007.

50. Moore GF, Audrey S, Barker M, et al. Process evaluation of complex interventions: medical Research Council guidance. *BMJ* 2015;350:h1258.

51. Cane J, O'Connor D, Michie S. Validation of the theoretical domains framework for use in behaviour change and implementation research. *Implementation Science : IS*. 2012; **7**: 37.

52. Glasgow RE, Vogt TM, Boles SM. Evaluating the public health impact of health promotion interventions: the RE-AIM framework. *Am J Public Health* 1999;89:1322–7.

53. Rochester CL, Vogiatzis I, Holland AE, Lareau SC, Marciniuk DD, Puhan MA, Spruit MA, Masefield S, Casaburi R, Clini EM, Crouch R, Garcia-Aymerich J, Garvey C, Goldstein RS, Hill K, Morgan M, Nici L, Pitta F, Ries AL, Singh SJ, Troosters T, Wijkstra PJ, Yawn BP, ZuWallack RL; ATS/ERS Task Force on Policy in Pulmonary Rehabilitation. An Official American Thoracic Society/European Respiratory Society Policy Statement: Enhancing Implementation, Use, and Delivery of Pulmonary Rehabilitation. *Am J Respir Crit Care Med* 2015;192(11):1373-86.

54. Burge AT, Holland AE, McDonald CF, Abramson MJ, Hill CJ, Lee AL, Cox NS, Moore R, Nicolson C, O'Halloran P, Lahham A, Gillies R, Mahal A. Home-based pulmonary rehabilitation for COPD using minimal resources: An economic analysis. *Respirology* 2020;25(2):183-190.

For peer review only

For peer review only - http://bmjopen.bmj.com/site/about/guidelines.xhtml

798 Table 1: Schedule of trial follow-up and procedures

Assessment/ Procedure	Baseline	End rehabilitation program	Monthly phone calls	12 months post rehabilitation program
**Informed consent**	X			
**Demographic information**	X			
**Program completion**		X		
**6-minute walk test**	X	X		X
**Dyspnoea-12**	X	X		X
**Health related quality of life (EQ-5D-5L and CRQ)**	X	X		X
**Physical activity participation**	X	X		X
**Healthcare utilisation**			X	X
**Economic evaluation**			X	X

799 CRQ – chronic respiratory questionnaire.

For peer review only

Table 2. Evaluation of implementation using the RE-AIM (Reach, Effectiveness, Adoption, Implementation, and Maintenance) framework

RE-AIM element	Quantitative and qualitative data
**Reach**	Intervention participation rates (number who chose home-based and centre-based rehabilitation)
**Effectiveness**	Program completion (>70% sessions attended), clinical outcomes
**Adoption** (further explored using the TDF)	Barriers and facilitators to implementation
**Implementation**	Program components delivered (e.g. exercise training/progression), use of exercise diaries
**Maintenance** (further explored using the TDF)	Intent to continue offering program choice, modifications made

TDF – Theoretical Domains Framework

Figure 1: Design of the HomeBase2 trial.

For peer review only

For peer review only - http://bmjopen.bmj.com/site/about/guidelines.xhtml

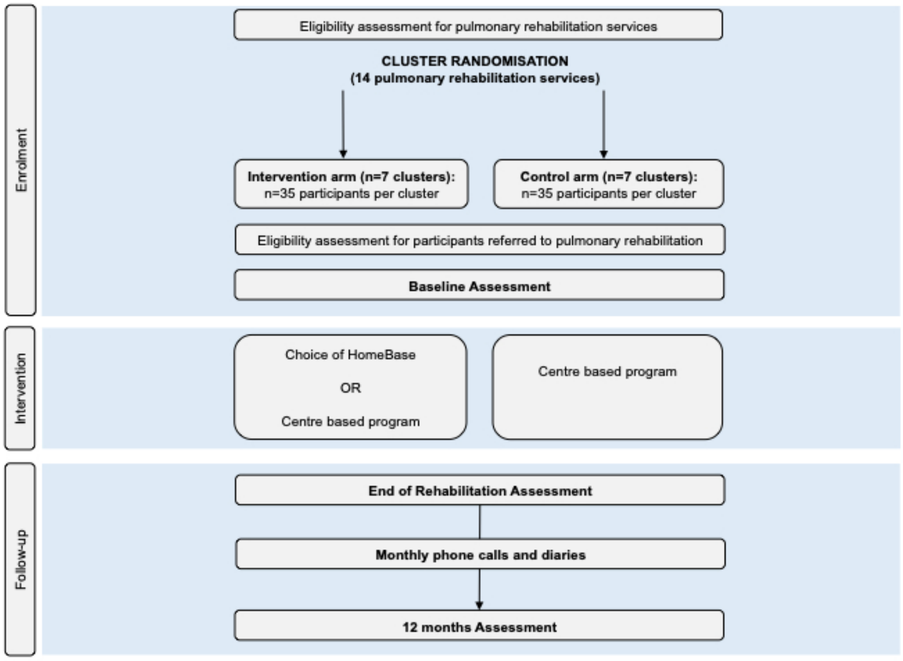

Figure 1: Design of the HomeBase2 trial

254x190mm (72 x 72 DPI)

STANDARD PROTOCOL ITEMS: RECOMMENDATIONS FOR INTERVENTIONAL TRIALS

SPIRIT 2013 Checklist: Recommended items to address in a clinical trial protocol and related documents*

Section/item	Item No	Description	Addressed on page number
**Administrative information**			
Title	1	Descriptive title identifying the study design, population, interventions, and, if applicable, trial acronym	_____1________
Trial registration	2a	Trial identifier and registry name. If not yet registered, name of intended registry	_____3________
	2b	All items from the World Health Organization Trial Registration Data Set	Trial Register____
Protocol version	3	Date and version identifier	_____19_______
Funding	4	Sources and types of financial, material, and other support	_______1_______
Roles and responsibilities	5a	Names, affiliations, and roles of protocol contributors	_______1______
	5b	Name and contact information for the trial sponsor	_______1_______
	5c	Role of study sponsor and funders, if any, in study design; collection, management, analysis, and interpretation of data; writing of the report; and the decision to submit the report for publication, including whether they will have ultimate authority over any of these activities	_______1______
	5d	Composition, roles, and responsibilities of the coordinating centre, steering committee, endpoint adjudication committee, data management team, and other individuals or groups overseeing the trial, if applicable (see Item 21a for data monitoring committee)	_______20-22____

For peer review only

Introduction

Background and rationale	6a	Description of research question and justification for undertaking the trial, including summary of relevant studies (published and unpublished) examining benefits and harms for each intervention	______4-7______
	6b	Explanation for choice of comparators	________6______
Objectives	7	Specific objectives or hypotheses	______7______
Trial design	8	Description of trial design including type of trial (eg, parallel group, crossover, factorial, single group), allocation ratio, and framework (eg, superiority, equivalence, noninferiority, exploratory)	________8______

Methods: Participants, interventions, and outcomes

Study setting	9	Description of study settings (eg, community clinic, academic hospital) and list of countries where data will be collected. Reference to where list of study sites can be obtained	________8______
Eligibility criteria	10	Inclusion and exclusion criteria for participants. If applicable, eligibility criteria for study centres and individuals who will perform the interventions (eg, surgeons, psychotherapists)	________8 & 9___
Interventions	11a	Interventions for each group with sufficient detail to allow replication, including how and when they will be administered	_______10-13____
	11b	Criteria for discontinuing or modifying allocated interventions for a given trial participant (eg, drug dose change in response to harms, participant request, or improving/worsening disease)	_________N/A___
	11c	Strategies to improve adherence to intervention protocols, and any procedures for monitoring adherence (eg, drug tablet return, laboratory tests)	_____15________
	11d	Relevant concomitant care and interventions that are permitted or prohibited during the trial	_______10-12____
Outcomes	12	Primary, secondary, and other outcomes, including the specific measurement variable (eg, systolic blood pressure), analysis metric (eg, change from baseline, final value, time to event), method of aggregation (eg, median, proportion), and time point for each outcome. Explanation of the clinical relevance of chosen efficacy and harm outcomes is strongly recommended	_______14-16____
Participant timeline	13	Time schedule of enrolment, interventions (including any run-ins and washouts), assessments, and visits for participants. A schematic diagram is highly recommended (see Figure)	14, Table 1 & Figure 1

For peer review only - http://bmjopen.bmj.com/site/about/guidelines.xhtml

Sample size	14	Estimated number of participants needed to achieve study objectives and how it was determined, including clinical and statistical assumptions supporting any sample size calculations	_____16-17____
Recruitment	15	Strategies for achieving adequate participant enrolment to reach target sample size	_______10______
**Methods: Assignment of interventions (for controlled trials)**			
Allocation:			
Sequence generation	16a	Method of generating the allocation sequence (eg, computer-generated random numbers), and list of any factors for stratification. To reduce predictability of a random sequence, details of any planned restriction (eg, blocking) should be provided in a separate document that is unavailable to those who enrol participants or assign interventions	_____8-9______
Allocation concealment mechanism	16b	Mechanism of implementing the allocation sequence (eg, central telephone; sequentially numbered, opaque, sealed envelopes), describing any steps to conceal the sequence until interventions are assigned	____8-9_______
Implementation	16c	Who will generate the allocation sequence, who will enrol participants, and who will assign participants to interventions	_____8-9______
Blinding (masking)	17a	Who will be blinded after assignment to interventions (eg, trial participants, care providers, outcome assessors, data analysts), and how	_____9________
	17b	If blinded, circumstances under which unblinding is permissible, and procedure for revealing a participant's allocated intervention during the trial	_______N/A______<br>_
**Methods: Data collection, management, and analysis**			
Data collection methods	18a	Plans for assessment and collection of outcome, baseline, and other trial data, including any related processes to promote data quality (eg, duplicate measurements, training of assessors) and a description of study instruments (eg, questionnaires, laboratory tests) along with their reliability and validity, if known. Reference to where data collection forms can be found, if not in the protocol	_____14-16_____
	18b	Plans to promote participant retention and complete follow-up, including list of any outcome data to be collected for participants who discontinue or deviate from intervention protocols	______14______

Data management	19	Plans for data entry, coding, security, and storage, including any related processes to promote data quality (eg, double data entry; range checks for data values). Reference to where details of data management procedures can be found, if not in the protocol	______21_______
Statistical methods	20a	Statistical methods for analysing primary and secondary outcomes. Reference to where other details of the statistical analysis plan can be found, if not in the protocol	______17-18____
	20b	Methods for any additional analyses (eg, subgroup and adjusted analyses)	______17_______
	20c	Definition of analysis population relating to protocol non-adherence (eg, as randomised analysis), and any statistical methods to handle missing data (eg, multiple imputation)	________17______
**Methods: Monitoring**			
Data monitoring	21a	Composition of data monitoring committee (DMC); summary of its role and reporting structure; statement of whether it is independent from the sponsor and competing interests; and reference to where further details about its charter can be found, if not in the protocol. Alternatively, an explanation of why a DMC is not needed	________20-21___
	21b	Description of any interim analyses and stopping guidelines, including who will have access to these interim results and make the final decision to terminate the trial	______N/A_____
Harms	22	Plans for collecting, assessing, reporting, and managing solicited and spontaneously reported adverse events and other unintended effects of trial interventions or trial conduct	______20-21____
Auditing	23	Frequency and procedures for auditing trial conduct, if any, and whether the process will be independent from investigators and the sponsor	______N/A______ _
**Ethics and dissemination**			
Research ethics approval	24	Plans for seeking research ethics committee/institutional review board (REC/IRB) approval	_____19-20_____
Protocol amendments	25	Plans for communicating important protocol modifications (eg, changes to eligibility criteria, outcomes, analyses) to relevant parties (eg, investigators, REC/IRBs, trial participants, trial registries, journals, regulators)	_____19________

Consent or assent	26a	Who will obtain informed consent or assent from potential trial participants or authorised surrogates, and how (see Item 32)	_____20__________
	26b	Additional consent provisions for collection and use of participant data and biological specimens in ancillary studies, if applicable	_______N/A_______
Confidentiality	27	How personal information about potential and enrolled participants will be collected, shared, and maintained in order to protect confidentiality before, during, and after the trial	_______21________
Declaration of interests	28	Financial and other competing interests for principal investigators for the overall trial and each study site	_________1_______
Access to data	29	Statement of who will have access to the final trial dataset, and disclosure of contractual agreements that limit such access for investigators	_______21________
Ancillary and post-trial care	30	Provisions, if any, for ancillary and post-trial care, and for compensation to those who suffer harm from trial participation	_________N/A_____
Dissemination policy	31a	Plans for investigators and sponsor to communicate trial results to participants, healthcare professionals, the public, and other relevant groups (eg, via publication, reporting in results databases, or other data sharing arrangements), including any publication restrictions	__________20_____
	31b	Authorship eligibility guidelines and any intended use of professional writers	_________20_______
	31c	Plans, if any, for granting public access to the full protocol, participant-level dataset, and statistical code	_______N/A_______
**Appendices**			
Informed consent materials	32	Model consent form and other related documentation given to participants and authorised surrogates	___Not provided
Biological specimens	33	Plans for collection, laboratory evaluation, and storage of biological specimens for genetic or molecular analysis in the current trial and for future use in ancillary studies, if applicable	______N/A_______

*It is strongly recommended that this checklist be read in conjunction with the SPIRIT 2013 Explanation & Elaboration for important clarification on the items. Amendments to the protocol should be tracked and dated. The SPIRIT checklist is copyrighted by the SPIRIT Group under the Creative Commons "Attribution-NonCommercial-NoDerivs 3.0 Unported" license.
